# A Review of Pressure Drop and Mixing Characteristics in Passive Mixers Involving Miscible Liquids

**DOI:** 10.3390/mi15060691

**Published:** 2024-05-24

**Authors:** Arijit Ganguli, Viraj Bhatt, Anna Yagodnitsyna, Dipak Pinjari, Aniruddha Pandit

**Affiliations:** 1 School of Engineering and Applied Sciences, Ahmedabad University, Ahmedabad 380009, India; virajpbhatt@gmail.com; 2 Institute of Chemical Technology, Mumbai 400019, India; dpinjari@gmail.com (D.P.); dr.pandit@gmail.com (A.P.); 3 Kutateladze Institute of Thermophysics, 630090 Novosibirsk, Russia; yagodnitsinaaa@gmail.com

**Keywords:** passive micromixers, computational fluid dynamics (CFD), pressure drop, mixing index, friction factor, regime map, design optimization, mixing

## Abstract

The present review focuses on the recent studies carried out in passive micromixers for understanding the hydrodynamics and transport phenomena of miscible liquid–liquid (LL) systems in terms of pressure drop and mixing indices. First, the passive micromixers have been categorized based on the type of complexity in shape, size, and configuration. It is observed that the use of different aspect ratios of the microchannel width, presence of obstructions, flow and operating conditions, and fluid properties majorly affect the mixing characteristics and pressure drop in passive micromixers. A regime map for the micromixer selection based on optimization of mixing index (MI) and pressure drop has been identified based on the literature data for the Reynolds number (*Re*) range (1 ≤ *Re* ≤ 100). The map comprehensively summarizes the favorable, moderately favorable, or non-operable regimes of a micromixer. Further, regions for special applications of complex micromixer shapes and micromixers operating at low *Re* have been identified. Similarly, the operable limits for a micromixer based on pressure drop for *Re* range 0.1 < *Re* < 100,000 have been identified. A comparison of measured pressure drop with fundamentally derived analytical expressions show that Category 3 and 4 micromixers mostly have higher pressure drops, except for a few efficient ones. An MI regime map comprising diffusion, chaotic advection, and mixed advection-dominated zones has also been devised. An empirical correlation for pressure drop as a function of Reynolds number has been developed and a corresponding friction factor has been obtained. Predictions on heat and mass transfer based on analogies in micromixers have also been proposed.

## 1. Introduction

Micromixers are devices or systems used to precisely mix fluids at the microscale level. The main purpose of micromixers is to efficiently blend different liquids or solutions, facilitating chemical reactions (Hessel et al. [1]), diffusion processes, or sample preparation. Microfluidic systems are used broadly in process engineering (Ganguli and Pandit [2]; Ganguli et al. [3]), bioengineering (Cai et al. [4]), microelectronics, and molecular chemistry and biology (Lee et al. [5]). The role of microfluidic systems is gaining attention, especially in chemical synthesis and biological diagnosis. Biological diagnoses include drug delivery (Khan et al. [6]) and microscale diagnostics, while chemical synthesis and associated applications include sample preparation for chemical analysis, for fast reactions, exothermic reactions, gas absorption, emulsification, foaming, blending, etc. Some of the key advantages include the fastness of mixing, with mixing times of less than 1 s, and suitability to laminar flow regime. Further, the mixing of small volumes in chemical and biological analyses ensures both mechanical and thermal safety. Compared with large-scale mixing devices, microfluidic systems offer advantages in terms of lower sample consumption, cheaper manufacturing cost, and higher throughput. 

Micromixing is generally achieved by two different types of micromixers: active micromixers and passive micromixers. Active micromixers utilize various forces including pulse-induced forces, electrostatic forces, magnetic forces, and acoustic forces. The active micromixers have been found to achieve a high mixing characteristic but they also incur an increase in the cost of mixing. Due to their higher costs, they are less preferable compared with the passive micromixers. On the other hand, passive micromixers involve the confluence of fluid streams with or without modifications in the mixing channel to enhance the mixing characteristics.

As passive micromixers only incur a one-time fixed cost of geometry fabrication and provide effective mixing, they are found to be useful in several fields of applications including but not limited to lab-on-a-chip [7,8], reaction engineering [9,10,11,12,13], medication diagnosis [14,15,16], and DNA analysis [17,18]. Passive mixing takes place by mixing where the energy input is by pumping power with channel arrangements in the form of split and recombine channels (SAR), chaotic mixing by eddy formation in T-junctions or other complex shapes, the nozzle injection of flow, or the collision of jets. Since the power required is directly proportional to the pressure drop, an optimum between the cost of energy needed to achieve the corresponding increase in mixing efficiency is vital. Hence, an estimate of the amount of energy required for a 1% rise in mixing efficiency is essential for every new micromixer design in addition to the novelty in terms of geometric shape and size of the micromixer. 

Research on passive mixers has been performed mostly using experimental work or numerical (mostly Computational Fluid Dynamics (CFD)-based) simulations. A review by Ngyuen et al. [19] was the first of its kind to have the nomenclature of active and passive mixers in which the authors also described the fabrication technologies and the techniques for performance evaluation. The dimensionless numbers that play a key role in understanding the various different aspects of fluid flow include (1) the effect of operating and geometric parameters given by Reynolds (*Re* = *d_h_uρ*/*μ*) *u* is the linear velocity and Capillary numbers (*Ca* = *μu*/*σ*), (2) ensuring that the continuum hypothesis in the system holds good given the Knudsen number *Kn* = λ/*L*, where *L* is the gap/characteristic length and λ is mean free path, (3) understanding if there were dean vortices and their influence on mixing given that the Dean number De=RedhRc is the radius of the curvature of the path of the channel and *d_h_* is the diameter for the circular cross-section and the hydraulic diameter for non-circular cross-section, (4) understanding the effect of Kelvin–Helmotz instability for wavy surfaces given that the Richardson number (*Ri*) is the ratio of buoyancy to inertial forces (*Ri* = ∆*bL/(*∆*U)*^2^) where ∆*b* is the buoyancy difference while ∆*U* is the velocity difference in a stratified layer, and (5) understanding the role of convection-diffusion on mixing given by the Peclet number (*Pe*). Nguyen et al. [19] posited that mixing with chaotic advection does not depend on the Peclet number (*Pe*). The Peclet number is the ratio of convection to diffusion given by (*Pe* = *uL/D_AB_*) where *u* is the linear velocity, *L* is the characteristic length, and *D_AB_* is the diffusivity of species ‘A’ diffusing in species ‘B’. The combination of *Re* and *Pe* numbers was responsible for understanding whether the mixing phenomena were based on diffusion, convection, or chaotic motion. For example, *Re* = 10 and *Pe* = 100 showed diffusion dominance while *Pe* = 1000 showed convection dominance. It is therefore imperative to understand that the concept of chaotic mixing is complex and needs special attention. A brief explanation of how chaotic mixing has been presented analytically is presented in brief. Extensive mathematical analyses have been suggested by researchers [20,21,22], concluding that fluid particles traverse different trajectories (termed as orbits) namely elliptic, hyperbolic, or parabolic periodic orbits. Another important method of quantification is in the form of Lyapunov exponents, which are numbers associated with orbits that describe the growth rate of infinitesimal perturbation. Chaotic flows have orbits with positive Lypunov exponents. Micromixer designs and microfluidic systems involving chaotic mixing more than 3 decades ago were designed on the basis of linked twist maps (LTMs). LTMs provided an analytical framework to design micromixers and satisfy the main conditions of mixing which were the squeezing, stretching, and folding of the fluid.

**Figure 1:**
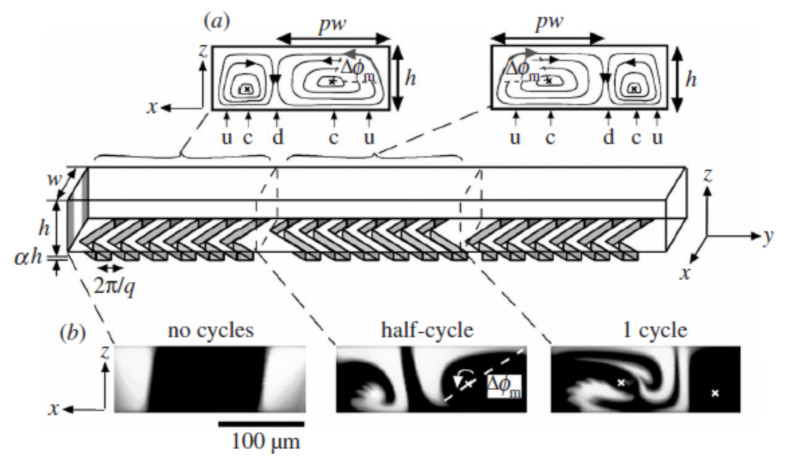
Closed streamlines in cross-section of each half cycle: (**a**) the mixer; (**b**) flow visualization in the cross-section (reproduced with permission from Wiggins and Ottino [20]).

Figure 1 shows the flow patterns of a typical micromixer with a straight channel with ridges on one of the walls arranged periodically to give a herringbone pattern. These ridges are responsible for giving rise to transverse flow. Each period consists of two half cycles producing two cells. In Figure 1a, streamlines are obtained analytically, while in Figure 1b, they are experimental photographs. In both cases, it can be observed that the cells are asymmetric and the elliptic points switch positions. Figure 1b shows the map between two half cycles and a map between two full cycles gives rise to LTMs. The cross marks on the photograph depicting 1 cycle represents the elliptic points.

Figure 1a shows the counter-rotating asymmetric cells, which are also termed as dipolar vortices by some researchers [23]. One needs to also remember that most numerical analyses consider no-slip walls on the lateral side and the asymmetric counter rotating rolls collide with the walls or two cells may collide head on with each other. During these collisions, kidney-shaped structures are formed temporally at the front and rear of the rolls and are quantified by the vertical and horizontal vorticity. More specifically, when a counter-rotating roll collides with a lateral wall, the vorticity (mostly the vertical component) increases, while without lateral walls, the vorticity values are zero. 

Having understood the basics of flow field in terms of vorticity and mixing we now try to understand the studies on classification and pressure drop in brief. Some researchers [1,4,5,24,25] defined the classification of active and passive mixers based on Re, mixing efficiency (η), and mixing length (*L_m_*). The classification was based on micromixer shapes namely (i) spiral based, (ii) overbridge based and (iii) chamber based. These depended on the fabrication and level of complexity. The amount of research performed on micromixer design is sufficiently large, resulting in this review of the literature on various micromixer designs and their performance being performed at an interval of very few years. Recently, a review on a selection of micromixers for different applications was carried out by Raza et al. [25]. The studies in this review were different than other reviews due to the fact that different geometrical shapes and sizes were used while keeping the *Re* constant. The researchers performed numerical studies for micromixers operating at identical operating conditions. The working fluids, Reynolds number, and axial lengths were kept identical. The authors presented a comprehensive approach for studies on pressure drop, undertaking simulation studies for 10 different geometries under five different mixing mechanisms based on the importance of inertial forces, turbulence, dean vortices, and multilamination. The studies covered an *Re* number range of 0.01 to 120 (*Re* = 0.01, 0.1, 1, 20, 40, 60, 80, and 120). The authors have chosen three *Re* ranges, namely, low range (0.01 < *Re* < 1), medium range (20 < *Re* < 40), and high range (60 < *Re* < 120). However, the pressure drop and mixing were compared for one particular Reynolds number *Re* = 10. A particular design that provided an optimum between pressure drop and mixing was recommended for several applications.

The literature review has shown that reviews on micromixers have focused on collating and analyzing the complexities in design, flow physics, and fabrication strategies. A few of the research works have also focused on particular micromixer designs and have evaluated either the MI or pressure drop, or both, for different Reynolds numbers. Some authors have estimated the costs involved based on the power needed to pump the fluid in micromixers, which depends on pressure drop. However, holistically, some questions need attention. For example, numerous micromixer designs are available in the published literature for different real-world applications. But a standardized nomenclature or abbreviation is not available for any micromixer. Further, the physics of mixing is well studied. However, there have not been regime maps for mixing indices. Further, a generalized and optimized region of operation for micromixers is not available. The present review attempts to address the above issues by defining the below objectives: 

(1) to develop a nomenclature for passive mixers based on special characteristics; (2) to devise design criteria for micromixers based on diffusion, convective advection, and chaotic motion-based processes; (3) to devise a selection procedure for passive micromixers on the basis of mixing index versus pressure drop; and (4) to propose correlations for pressure drop based on above data. In the present review, liquid–liquid miscible systems are analyzed. The immiscible systems referred to are restricted to presenting different geometrical shapes, which can be used for carrying out mixing studies for miscible systems.

The review is organized as follows: In Section 2, a categorization of micromixers is performed based on the construction of micromixers. In this effort, micromixers that have been reported in the literature in the last two decades (96 micromixers) have been categorized based on their complexity into four different types. Further, the names of each micromixer and its abbreviation (as per authors’ terminology if available or defining a terminology in present work) have been provided. Since the reviews by researchers [25,26] comprise all the micromixers that underwent experimental and numerical investigations, most of the 96 micromixers reported in the present work for categorization consist of micromixers that have been reported in the published literature (focusing on research undertaken in the last 5 years). After summarizing the experimental and numerical investigations, the analytical expressions for pressure drop and mixing index are collated and presented in Section 3 and Section 4. In Section 5, a comprehensive discussion on how the collated pressure drop and MI data can be used for the selection of micromixers has been designed. In Section 6, the correlations for friction factor and transport parameters like heat and mass transport coefficients based on analogies have been derived from the pressure drop data.

## 2. Categorization of Passive Micromixers

The geometric features of the microfluidic system play a crucial role in the micromixing of fluids, which in turn influences the transport phenomena like mass and heat transfer. When in a passive micromixer, fluid streams contact each other, creating an increase in pressure drop across the microchannel. The pressure drop is a crucial aspect in the design considerations. A detailed discussion on the pressure drop of the passive micromixers will be undertaken in the forthcoming sections. Hence, optimization in terms of pressure drop and mixing is necessary. 

The categorization of the micromixers are termed as (1) Category 1 or simple passive micromixers, (2) Category 2 or passive micromixers with flow direction variation, (3) Category 3 or passive micromixers with flow obstructions, and (4) Category 4 or complex passive micromixers, respectively. The basis for the categorization and description for each category is provided in brief in the forthcoming paragraphs. 

In Category 1, the mixing takes place at the junction and fluid moves to the main channel. Mixing is mainly via diffusion and a low Reynolds number. Further, the dimensions of the channels, such as width, height, and length, influence the fluid dynamics and mixing efficiency. Smaller channel dimensions typically result in higher mixing and enhanced diffusion at the cost of an increased pressure drop. The aspect ratio, which is the ratio of channel width to height, can impact mixing performance. Higher aspect ratios (i.e., narrower channels with greater height) tend to promote lateral mixing due to increased shear forces and flow interactions, leading to improved mixing and higher MI values [27]. Several strategies apart from channel dimensions include changing the configuration of the inlets through which the fluids enter the micromixer. Varying the inlet positions, angles, or introducing asymmetry, can induce vortices, swirls, and cross-stream mixing, leading to enhanced mixing performance. The design of the micromixer, including its geometry and channel layout, can significantly impact the mixing index irrespective of the category of the micromixer [27]. In Category 2, mixing takes place due to changes in flow direction by chaotic intermixing along with diffusion. These are easy to construct and fabricate and mostly rely on dean vortices for efficient mixing. This implies that the flow depends on two dimensionless numbers, namely the Reynolds number and the Dean number [28]. Micromixer channels with curved or tortuous geometries can enhance mixing by inducing secondary flows, or the stretching and folding of fluid streams [29,30]. Curvature and tortuosity introduce additional fluid mixing mechanisms, resulting in improved mixing efficiency and a higher mixing index. Tapering the channel width or height along its length can affect fluid flow and mixing. Gradually narrowing or widening the channel can create pressure gradients, induce flow instabilities, and generate vortices, which enhance mixing.

In Category 3, passive micromixers, mixing chambers, grooves [31], or obstacles [32,33,34,35] within the channels can induce the chaotic advection, stretching, folding, and splitting of fluid streams, leading to enhanced mixing. The overall connectivity and arrangement of channels within a micromixer can influence mixing performance. 

In Category 4, designs incorporate interconnected networks [36] or hierarchical structures [37] into hybrid mixers [38,39] that incorporate obstacles and curvature. These can facilitate multi-stage mixing and chaotic mixing and increase residence time, thereby improving the mixing index. Further, these micromixers exhibit chaotic flows (Ruijin et al. [40]). In such flows, the flow occurs in orbits and are measured by the Lypunov exponent, which is a number associated with the orbit of a spiral shape describing its stability in the linear approximation. These flows are characterized by positive values for the Lypunov exponent.

Table 1 comprises the studied geometries and categorization of these geometries into the above four categories. Although different authors have classified active and passive micromixers with a typical nomenclature, it was thought worthwhile to list the names and their abbreviations before categorizing them into types 1 to 4. Table 2 summarizes the most recent and significant experimental investigations on pressure drop, with mixing indices for different *Re* ranges mostly limited to less than 600. Table 3 summarizes the recent numerical studies by various researchers. The Peclet number and Reynolds number ranges of the authors, their geometrical dimensions, and the pressure drop and mixing indices have been represented. All the meshes used for grid sensitivity have been represented by notations M1 to M4 where M1 represents the lowest number of mesh elements while M4 represents the highest number of mesh elements. This affirms that all researchers have compared at least three mesh sizes for the grid sensitivity and arrived at the best mesh that gives the best results in terms of mixing. For example, Farahinia and Zhang [41] found that reporting grid sensitivities based on flow parameters gave erroneous mixing results. Hence, *Pe* number is very important to ensure that numerical diffusion does not show fictitious values of MI. For example, Farahinia and Zhang [41] found that the MI values predicted by the coarsest and the finest grid varied up to 22% over predictions due to numerical diffusion.

The most important aspect of Table 1 is the abbreviations defined for each micromixer in Column 3. For all categories, the abbreviation is represented in the form AB-ZZ-M where M represents the term “Micromixer”, ZZ represents the shape of the micromixer, and AB represents the specialty of the micromixer, like obstruction or an adjective for the shape like twisted or tangential. For example, in the case of a Category 1 micromixer like TJM, “ZZ” is TJ or T-junction. Similarly, for other categories, “SH” would represent “Staggered Herringbone” while “Sw” would represent “Swirl”. Further, for a Horizontally Split T-junction micromixer (HSTJM), AB represents HS; ZZ represents TJ. Similarly for other micromixers, AB may represent “Elliptical Obstructed”, “Rectangular Insert”, “Diamond Obstructed”, etc., as can be observed in the abbreviations in Table 1. AB may also represent three words, as in the case of the Pore Array Intensified Tube-in-Tube Micromixer, AB represents Pore Array Intensified, while ZZ represents Tube-in-Tube.

There might be some exceptions as to what some researchers [87] call specific micromixers. In such cases, the abbreviation has been modulated to accommodate the existing abbreviation. For example, for a micromixer named Asymmetrically Distributed Z-Shaped Baffled Micromixer (ASZMM), the abbreviation is modulated as AB representing ASZ while ZZ represents M, with the last letter M as Micromixer.

Table 2 presents various parameters used during experimental investigations by authors. Most authors [62,70,90] have considered a mixture of pure water and concentrated water, with the concentration being made up of dyes or agents like ink or methylene blue. 

Some researchers [69,87] have used a water–ethanol system while Mariotti et al. [91] used a methylene blue–ascorbic acid system. The investigations included in the table are mostly in the *Re* range of 0.1 to 100 with some researchers [70] having a maximum *Re* around 260 while some researchers [90] reached up to 400. The pressure drops reported were as low as a few pascals for the lowest *Re*, to considerable kPa’s (~50 kPa) for high *Re*, depending on micromixer design. The MI range is wide, in most cases ranging from 0.2 to 1, with a few exceptions [87] where the MI values are very high.

Table 3, on the other hand, shows the details of researchers carrying out numerical investigations. While the dimensions and systems used by the authors were similar to the ones described for experimental investigations, two major commercial pieces of software, namely Ansys Fluent and Comsol Multiphysics, have been used by most researchers, followed by OpenFoam by a few of them. The pressure drop range is similar to the researchers performing experimental investigations. However, a preliminary comparison of the category-wise micromixers based on the data of Table 3 shows that the Category 4 micromixers have a narrow range of MI values starting from 0.8, while Categories 2 and 3 have minimum values of 0.1 and 0.2, respectively. Although the corresponding upper limit of the pressure drop for the categories were 80, 45, and 9 kPa, respectively.

Thus, Table 2 and Table 3 provides us with not only the geometric and operating parameters but also with a preliminary estimate of the kind of pressure drops and MI values one can expect based on the category of micromixers.

### 2.1. Category 1: Simple Passive Micromixers

As discussed in Section 1, passive micromixers are easy to construct, typically having junctions in laminar conditions. The construction is achieved using microfabrication techniques such as micro-milling or soft lithography [19]. While these micromixers offer good mixing, they may be limited in mixing efficiency or control when compared with more complex micromixers [4]. However, they serve as fundamental building blocks for more advanced micromixers and provide valuable insights into the principles of microscale fluid mixing.

**Figure 2:**
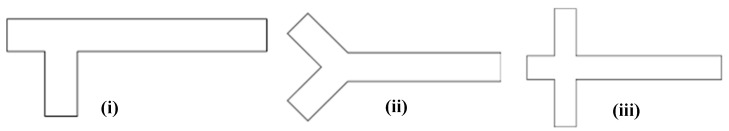
Various Types of Category 1 Passive Micromixers: (**i**) T Junction Mixer (TJM); (**ii**) Y Junction Mixer (YJM); (**iii**) Cross Flow Junction Mixer (CFJM).

The simple passive micromixers comprise micromixers with the fluids flowing in laminar conditions. Figure 2 shows the common conjunction geometries used for contacting the fluids at lower Reynolds numbers. These include the T-Junction Micromixer (TJM) (Figure 2i) and the Y-Junction Micromixer (YJM) (Figure 2ii) for the contact of two fluid phases and the cross flow junction micromixer (CFJM) (Figure 2iii) for the contact of three fluid phases. The fluids’ streams meet each other creating a conjunction (like T, Y or cross flow shape as shown in Figure 2i–iii). The fluids flow simultaneously with an interface between them. This type of flow is termed as ‘Stratified Flow’ [93]. The dominance of gravity forces on stratified flow is dependent on the height of the micromixer. Minakov et al. [93] have reported that below a channel height of 200 μm, the effect of gravity can be neglected, while above 300 μm, the effect of gravity should be taken into account. In such cases, the fluid phase heavier than the other settles at the bottom and flows along the lower part of the microchannel and the lighter fluid phase flows along the upper part of the microchannel with an interface between them. The interface between the fluid phases is continuous and of four major types, which include (1) smooth interface, (2) vortex flow, (3) non-stationary periodic flow, and (4) stochastic flow. A smooth interface is observed in microfluidic systems at lower Reynolds numbers (*Re* < 5) of both the fluid phases, also referred to as stationary vortex free flow by researchers Minakov et al. [93]. Vortex flows may be symmetric with two horseshoe-shaped vortices (5 < *Re* < 150) or asymmetric and stationary (150 < *Re* < 240). The nonstationary periodic flows (240 < *Re* < 400) and stochastic flows (*Re* > 400), however, seem to provide higher mixing. The mixing efficiencies are found to be highest for stationary vortex flows, according to Minakov et al. [93]. Strang and Fernando [94] reported that the transition from a smooth surface to a wavy surface was caused by Kelvin–Helmotz instability and characterized by the Richardson number (*Ri*). 

### 2.2. Category 2: Passive Micromixers with Flow Direction Variation

Based on the recent available literature, geometric designs consisting of either only meanders in the flow path or a combination of meanders and other shapes are included in this category. Further, the number of meanders are key to the mixing, since these are the prime parameters of the flow, characterized by secondary flows known as Dean vortices and given by the Dean number (De). Table 1 only lists the articles in this category with the name and abbreviation (either provided by the authors or specified by the present work for simplicity).

These passive micromixers with flow direction variation exploit the complex flow patterns and mixing mechanisms induced by changes in fluid trajectory. They offer advantages such as simplicity of design, ease of fabrication, and effectiveness in achieving mixing at the microscale. Optimization and customization may be required to achieve specific application-mixing outcomes. Continuous changes in flow direction leads to the formation of Dean vortices. The major difference between the flow in a straight channel compared with one in a curved channel is due to the fluid motion. This occurs due to centripetal forces and has been described by researchers [28,70] as follows: the presence of a curved structure (a turn) in the low path of the fluid causes the formation of a pressure gradient along the former side of the curve and the latter side of the curve. This pressure gradient leads to a velocity gradient along the curve. This leads to a secondary motion of the fluid in the form of a vortex with the primary fluid flow along the microchannel. The Dean vortex is the pair of these counter rotating vortices.

**Figure 3:**
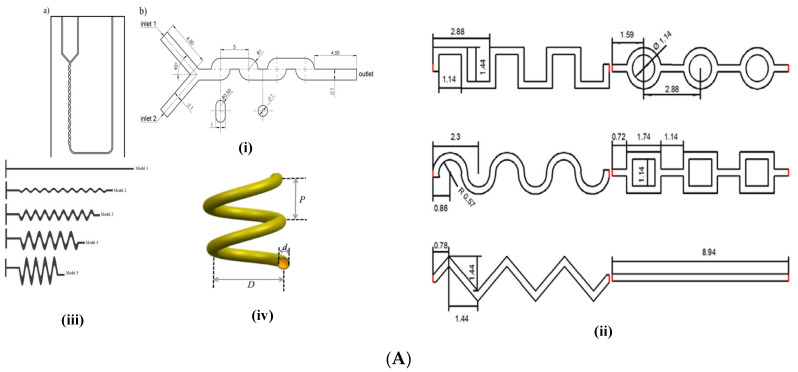

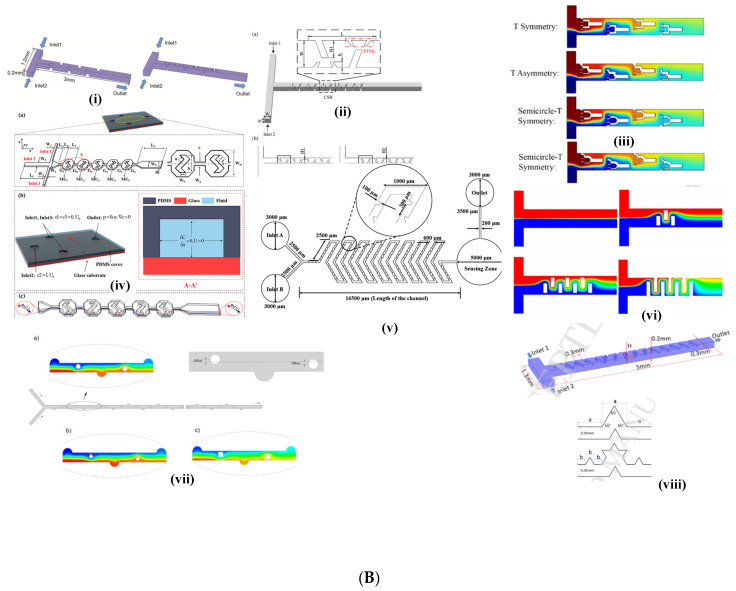

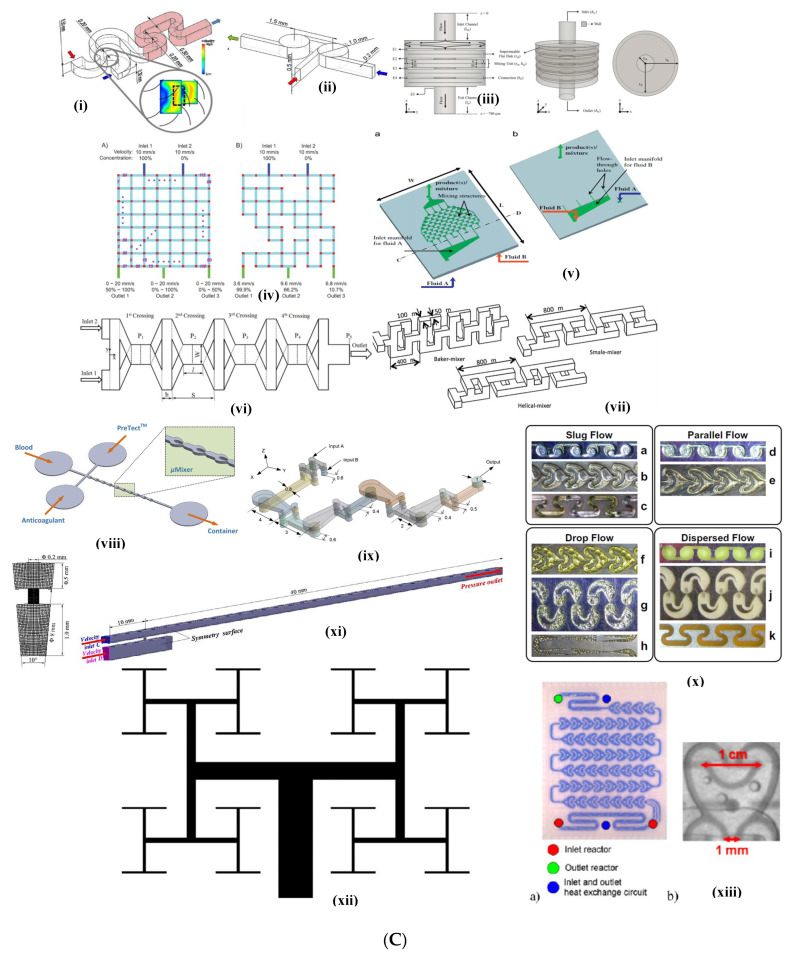
(**A**). Category 2 Micromixers: (i) LTFMS Micromixer Khaydarov et al. [70]; (ii) Sinusoidal, Trapezoidal, and Triangular Micromixer Chen et al. [65]; (iii) Sinusoidal micromixer, Parsa et al. [62]; (iv) Helical Coil Micromixer, Luo et al. [28]. (**B**). Category 3 Micromixers: (i) Primary and Secondary Minkowski Fractal Obstacle Micromixer, Chen and Chen [74]; (ii) Staggered Baffle Micromixer, Niu et al. [32]; (iii) T-type Fractal Obstacle Micromixer, Hou et al. [33]; (iv) ASZMM micromixer, Yuan et al. [87]; (v) Obstacle Serpentine Micromixer, Karthikeyan et al. [89]; (vi) T-type mixer with rectangular inserts, Rudyak and Minakov, [34]; (vii) Obstructed Grooved Micromixer, Rahmannezhad and Mirbozorgi, [31]; (viii) Primary and Secondary Koch Fractal Baffle Micromixer, Zhang et al. [35]. (**C**). Category 4 Micromixers: (i) Twisted Y-Junction mixer; (ii) Tangential T Junction, Fischer and Kockmann, [54]; (iii) Circular-shaped Fluid Overlapping Micromixer, Okuducu and Aral, [78]; (iv) Random mixers, Zhang et al. [37]; (v) Multi-laminational elongated flow mixer, Adeosun and Lawal [36]; (vi) Two-layer crossing microchannel micromixer, Amar et al. [77]; (vii) Helical, Baker, and Small Micromixers, Ruijin et al. [40]; (viii) SAR micromixer for blood mixing, Tran-Minh et al. [61]; (ix) HC Micromixer, Mahmud et al. [83]; (x) Sickle, venture, SZ-type micromixers, Plouffe et al. [38]; (xi) Pore Array Tube-in-Tube micromixer, Li et al. [13]; (xii) Tree Micromixer, Wang et al. [57]; (xiii) Corning Advanced Flow Reactor, Nieves-Remancha et al. [39].

The present section describes the different designs of Category 2 micromixers’ corresponding flow patterns and their advantages and challenges for implementation. Figure 3A shows the major types of the Category 2 micromixers. We explore one of the Category 2 designs [70] in detail to explain the terminology of Category 2 introduced in the present work. In one of the interesting investigations (both experimental and numerical), Khaydarov et al. [70] considered micromixers consisting of meander elements and recirculation chambers, which the authors named as S-form or chicanes (as shown in Figure 3B(i)). A micro photo camera was used for experimentation and Ansys CFX 14 for their numerical simulations. The geometry consisted of a 90° Y-junction Micromixer (YJM) with an inlet diameter of 1 mm. To elaborate, two cylindrical geometries were connected to each other using a 90° embedded elbow geometry. A single 90° elbow formed one meander element. The total length of the micromixer was 21 mm consisting of 22 such meander elements as shown in Figure 3A(i). An increase in mixing by increasing the number of meander elements was observed. A few other similar geometries are shown in Figure 3A [65,69,84]. Chen et al. [65], using experimental investigations, designed different types of micromixers, namely square wave, multiwave, and zigzag serpentine micromixers (Figure 3A(ii)) and found advantages and limitations in each of the designs. Research work on sinusoidal mixers has been carried out in detail by Parsa et al. [62] (Figure 3A(iii)), who showed the role of Dean vortices in improving MI. Similar results have been found for a helical coil mixer (Figure 3A(iv)) by Luo et al. [28], where De numbers are high and aid the best performance for Category 2 micromixers. Other designs for Category 2 include vortex micromixers [69] and spiral and serpentile micromixers [84]. The design of Ansari et al. [69] was simple in design since conventional T-junctions and vortex micromixers (micromixers similar to T-mixers which have the structure of an hydraulic jump) were investigated. These designs are characterized by circular rolls and kidney-shaped structures over horizontal and vertical walls, as described in Section 1 [23]. With the advent of numerical simulations and tools for Computational Fluid Dynamics, the analysis can be performed in a much more rigorous manner. The material of construction of these micromixers was Teflon. Integration of different micromixers also enhanced the mixing. For instance, the vortex mixer when integrated with serpentine microchannels gave high mixing performance with values as high as 5 times than that of simple micromixer designs were observed showing the significance of hybrid approach. Similar hybrid approaches have been carried out by other researchers [65,84]. Tripathi et al. [84] compared three different micromixers, spiral, serpentine, and straight microchannels, and found spiral micromixers to provide the best performance after a critical flow rate (*Re* = 25). The spiral micromixers can be better designed and understood from the fundamentals of LTMs and Lyapunov exponents and are characterized by the authors as sirl strength, which is nothing but vorticity. Although numerical simulations aid in an understanding of the physics and vorticity magnitudes in a detailed manner, it is always recommended to understand the physics of phenomena with the fundamentals provided by researchers [20,22]. This section highlights a few micromixer designs of Category 2 and that there is a big role to play for the operating and geometric parameters, which will be discussed in Section 3 and Section 4.

### 2.3. Category 3: Passive Micromixers with Flow Obstructions

Category 3-type micromixers have been found to be the most preferred category or design method for researchers, compared with the other types of micromixers considered in the present work. These micromixer designs are characterized by having different types of obstructions to facilitate good mixing. The designs have mostly been investigated via numerical simulations. We describe in brief the characteristics of innovative designs in this category by researchers [33,80,82], focusing on their size, shape, advantages, challenges faced in numerical simulations or fabrication, and applications. To maintain brevity, only a few designs have been shown in Figure 3B.

While most researchers have used obstacles in simple geometries like T-junctions or cross flow junctions, some have designed micromixers’ biomimicry of natural networked structures (for instance tree-like structures), while some have used herringbone structures. All designs, however, had obstacles of different shapes and sizes since flow obstructions facilitate in generating different flow patterns and turbulence, which, in turn, facilitate rapid and efficient mixing at the microscale. However, it is important to optimize the design parameters, such as the size, shape, and spacing of the flow obstructions, to achieve the desired mixing performance for specific applications. Hosseini and Rahimi [76] used a T-shaped micromixer with high length (100 mm long) with five different micromixers (refer to Table 1), each with four barriers of various shapes. The number of barriers differed with micromixer design. For example, in the case of one of the variants (the CoM micromixer; refer to Table 1), the number of barriers ranged from 4 to 7, while with other variants, the number was as high as 10. Okuducu and Aral [78] carried out numerical simulations in a T-junction micromixer using twelve semi-circular ridges that were convex in the streamwise direction. Typically, the dimensions of the ridges were related to the height and width. For example, the ridges were around half the height and three/fourths of the diameter of the channels while the thickness was one tenth of the height and width of the channels. The semi-circular ridges were aligned with a pitch length of 150 µm, starting from the confluence region. Silva Jr et al. [72] used a cross flow micromixer (referred to as Uniform Circular Cross Flow Micromixers (UCCFM)). Circular obstacles were positioned in an equilateral triangular arrangement with a pitch related to the obstacle diameter (three times obstacle diameter (3d_obs_)) and studies were carried out in the range 150 < d < 600 µm. The numerical simulations were carried out using two different liquid mixtures (water–oil and oil–ethanol) with rhombic cross flow micromixers comprising three inlets (one at the top and two at the corners) and an outlet at the bottom. Farahinia and Zhang [75] have carried out numerical simulations in simple T-junctions as well as T-junctions with vertically straight or inclined baffles (rectangular or diagonal barriers, furrows). As compared with simple T-junctions, these geometries were found to provide an increase in mixing efficiencies of up to 40–44% for the same mixing length, consisting of circular obstructions with triangular pitches across the entire rhombus. A similar approach of designing micromixers with obstacles was adopted by Niu et al. [32], using numerical simulations. A Category 3 micromixer with cross staggered baffles (CSBs), with paired trapezoidal microstructures, was designed (Figure 3B(ii)). Some designs were found to be biomimicked from nature, like tree-like networks, as observed in plant nutrition, or mammalian blood circulation, or fractals. For example, Hou et al. [33] used tree-shaped fractal or bifurcation networks in T-junction geometries based on Murray’s law (which is one of the basic laws of branch structures) which could be applied to non-living systems. Several variations in the shape, type of the geometry, and obstruction were incorporated, such as six different shapes of fractals (flat, semi-circular), different fractal tip angles, and two different methods of placement methodologies, namely symmetrical and asymmetrical. (refer to Figure 3B(iii)). The authors varied their geometric parameters like width, length, height, diameter, and cross-sectional area, etc. The main advantage of such designs lies in their ability to operate at very high efficiencies (refer to Table 2). Chen and Chen [74] carried out simulations with Minkowski fractal obstacles in T-junction micromixers (Figure 3B(i)) with two variants, namely (i) a primary variant with a shape similar to a step input and (ii) a secondary variant with two fractals. The first step had the size L while the second had the size L/3 and the third had size L/9. Talebjedi et al. [80] designed a T-shaped micromixer with deformable obstacles using numerical simulations. This design requires special mention due to the challenges in modeling the dynamics of deformation using moving boundaries and moving meshes using the Arbitrary Lagrangian Eulerian (ALE) method in COMSOL Multiphysics (version 5.4). The major advantage of such designs was the change in the track of the fluid with obstacle deformation. Further, the design of experimental techniques like the Taguchi algorithm were used to optimize design variables and number of simulations. Another variant of a Category 3 micromixer design can be found in the design by Wang et al. [82], who used numerical simulations using the commercial software Comsol Multiphysics (version 5.5A) (referred to as Tesla micromixer with diamond obstacles). The design consisted of a horizontal main channel that had inclined U shapes on either side of the channel and diamond shaped obstacles near the entrances. Yuan et al. [87] designed a micromixer that consisted of asymmetrically distributed Z-shaped baffles with alternated positive and negative values on the Y-axis (refer to Figure 3B(iv)). The design differed from other micromixer geometries by having a repeated arrangement of obstacles and was responsible for producing a chaotic effect and promoting asymmetric flow. The authors termed this mixer as an Asymmetry-distributed Z-shaped Baffle Micromixer (ASZMM) and was targeted for its use in biochemical applications. Karthikeyan et al. used a Y-shaped herringbone-shaped micromixer with obstacles (Refer Figure 3B(v)). Since herringbone-shaped micromixers have been described in other reviews, a detailed description of the design has not been provided in the present review. Zhang et al. [35] numerically studied mixing characteristics in a Primary Koch Fractal Baffle Micromixer (PKFBM) and Secondary Koch Fractal Baffle Micromixer (SKFBM) (Figure 3B(viii)). The geometry consisted of as large as fifteen fractal baffles in total. The PKFBM consisted of a symmetrical triangular baffle while the SKFBM consisted of spike-like geometries over the symmetrical geometry of PKFKM. Borgohain et al. [71] carried out numerical studies in three types of microchannels: 1. Cross T-junctions with curved obstacles (the design can be compared with an aerofoil design). The obstacles were placed in pairs consecutively on the top and bottom of each obstacle in a pair, one half of the pair being a mirror image of the other (the authors call it a Winglet pair). Since the authors performed numerical simulations, it was assured that the Kundsen number was less than 10^−3^. A combination of this design with different designs like the Rhombic micromixer (Chung and Shih [95]) and microchannels with LVG (Hsiao et al. [96]) were designed, which provided a 2–5 times increase in mixing performance when compared with their individual micromixer design. Further, the design also provided an optimized pressure drop that had the least energy consumption. For achieving this objective, a comprehensive sensitivity of the geometric parameters like angle, height, number of obstacles, and operating parameters like velocity (represented in *Re*) were performed.

### 2.4. Category 4: Complex Passive Micromixers

As discussed, in earlier sections, Category 4 micromixers are complex passive micromixers and advanced microfluidic devices that incorporate intricate designs and multiple mixing principles to achieve highly efficient and controlled mixing. These micromixers often combine various features such as flow obstructions, flow splitting or merging, and flow recirculation to produce secondary flows. They also induce transition from laminar flow to turbulence and enhance the mixing performance. In this subsection, some of the selected designs of micromixers reported in the literature have been discussed. Innovative Y and T junction mixers have been designed by Fischer and Kockmann [54] (Figure 3C((i) and (ii)). Okuducu and Aral [78] designed the circular shaped fluid overlapping Micromixer while another innovative design is the Random mixers presented by Zhang et al. [37]. Adeosun and Lawal [36] designed a Multilaminated Elongational Flow Micromixer (MEFM) (Figure 3C(v)) which consists of a manifold which, in turn, consists of several trapezoidal elements with the geometric focusing nature of the mixture design layout that imparts elongation flow via folding and the local and global re-orientation of fluid interfaces. The last five rows of the trapezoidal mixing structure were flipped to somewhat form a mirror image of six upstream rows. A numerical model was validated with experiments. Nieves Remancha et al. [97] used Corning micromixers, which consisted of series of spade-shaped structures with obstacles that disrupt the flow to form vortices and promote mixing (Figure 3C(xiii). Wang et al. [57] designed fractal micromixers without obstacles. The structure was developed by bio-mimicking from tree structures, similar to the structure of lungs or the vascular network of mammals or the structural network of the leaves of trees. In this mixer, every parent channel structure is divided into two daughter branches at the next level where single channel bifurcates with a bifurcating angle of 1800 (Figure 3C(xii)). Secondary flows cause rapid mixing stratified vortices characterized by the engulfment regime. Plouffe et al. [52] have used different curved shapes in the form of meandering, serpentine, and recirculating channels (which the authors termed SZ and TG). The authors also used other types of hybrid mixers like venture, Sickle, and spade for deriving secondary flow patterns (Figure 3C(x)). Tran-Minh et al. [61] designed a passive SAR micromixer with ellipse-like microcapillaries for blood flow (Figure 3C(viii)). A single unit of the micromixer consisted of a smaller ellipse inside two half ellipses of a bigger size. The entire micromixer was designed in a way in which the concept of SAR was utilized. During operation, the flow separation took place at the wall of the smaller ellipse (entry) into two separate flows, while at the end of the smaller ellipse and the junction of the merger of the half ellipses (exit), two separated flows were recombined with an increase in velocity compared with the entry. Typically, the micromixer consisted of 10 units and the mixing was enhanced with passage from unit 1 to 10 with the outlet consisting of almost well mixed liquid. Ruijin et al. [40] designed a splitting and merging structure in three ways and named them small, helical, and Baker mixer (Figure 3C(vii)). The Baker mixers were designed using Baker transformation (Ruijin et al. [40]) for low *Re* while the small and the helical mixers were SARs acting under a similar principle but with higher mixing and high *Re*. Amar et al. [77] designed a micromixer (Figure 3C(vi)) where the elongation of the crossing zone was repeated four times to make a micromixer unit. The designs were based on the variation of length to width ratio. Mahmud et al. [83] (Figure 3C(ix)) performed numerical simulations for a special type of mixer consisting of one Y- and one U-shaped segment connected by a cylinder. It was designed using the SAR principle.

To summarize, a considerable amount of research has been conducted for all categories of micromixers both in terms of experimentation and numerical simulations. Many authors have performed only numerical simulations for their design with validated models but have not performed experimentation by themselves. Various different designs in terms of geometric parametric variations have been created and operating parameters have been varied. Some researchers have covered both experimental and numerical aspects and validated the models with experimental data. Overall, it has been observed that in CFD simulations, (both pressure drop and MI data) the major parameter ensuring good agreement is the grid resolution and making sure that the *Pe* numbers are such that accurate MI predictions were possible with low deviation from experimental results in terms of MI values.

## 3. Mixing Index (MI) of Passive Micromixers

As discussed in Section 2, the effectiveness of a micromixer depends on how well the fluids are mixed together measured by MI. It also quantifies the degree of homogeneity achieved in the mixing process. The MI is typically determined by analyzing the concentration distribution of a tracer species within the fluid flow (Sommer [98]). MI depends mainly on *Re* and *Pe* numbers. Experimental investigations involve measuring concentrations of tracers for different operating parameters. CFD simulations involve solving the concentration species equation for two mixing fluids along with continuity and momentum equations.

### 3.1. Empirical/Analytical Expressions Used for Calculation of Mixing Index in Passive Micromixers

The measurement of MI depends on the standard deviation of the mixer and is represented based on the number of sampling points. Table 4 lists a few expressions for MI available in the literature

As depicted in Table 4, most of the researchers [52,55,99] provided the expressions in the form of standard deviation, which in turn are functions of concentrations of sampling points and the mean concentration. Some researchers [34] have expressed MI as an empirical expression which is a function of the Peclet number. The prominent feature of the correlations is that the mixing index has a non-linear relationship with the Peclet number. Other than the above discussed methodologies for the characterization of mixing, several other methodologies are used, which include RTD analysis, segregation index analysis, and analytical models based on Taylor dispersion theory, which is beyond the scope of this review.

### 3.2. Effect of Operating, Geometric Parameters, and Fluid Properties on Mixing Index in Passive Micromixers

Two important parameters profoundly affect the MI of the passive mixers, which are the flow rate (an operating parameter) and the diffusion coefficient (property of the fluid). The dimensional numbers associated with these parameters are *Re* and *Pe*. Optimized values for both parameters are required. For example, higher flow rates can enhance convective mixing and reduce the diffusion length scales, resulting in improved mixing. However, excessively high flow rates can also lead to non-uniform velocity profiles and inefficient mixing. Similarly, fluids with higher diffusion coefficients tend to mix more rapidly, while those with lower diffusion coefficients may require additional mechanisms, such as convective mixing, to achieve efficient mixing. Further, MI is a function of various parameters depending on the category of the passive mixer whether curvature in the flow path, the presence of obstacles or, the complexity of the overall design. MI increases happen across the axial length of most of the micromixers with the highest at the outlet of the mixer. Hence, axial variation in MI is very important for understanding the role of convection over diffusion with increases in *Re* and *Pe*.

The present section summarizes research works showing the effect of the above mentioned two dimensionless parameters on MI. This includes the effect of *Re* and *Pe* on MI performed by researchers for the different categories of micromixers discussed in the present work. In these works, the dimensions of the mixers, like the length, width, and height, are constant and only the fluid velocity is different. Further, a discussion on the comparison of the MI values obtained from different research works involving Categories 2, 3, and 4 mixers with Category 1 micromixers has been presented.

Khaydarov et al. [70] used a micromixer of S form integrated with a Y-junction micromixer and carried out a flow and mixing performance analysis for the Reynolds number range (0.1 ≤ *Re* ≤ 260). The system considered was a water–dye system. Some interesting observations on MI variation and flow patterns were observed. The MI values showed wide variation in the range (0.1 < MI < 0.85) while the stratified flow was observed for low *Re* (*Re* < 2.6) and vortex flow and Dean vortices were observed after *Re* = 13 to *Re* = 259. The type of variation was also interesting. For example, MI rose up to 0.75 for *Re* = 259 and was found to be a linear function of *Re* for the entire *Re* range (13 < *Re* < 250). The convective mixing significantly increased with *Re* due to the presence of the meander elements. Lobasov and Minakov [27] observed that in TJMs (Category 1 micromixer), with a water–dye system, an increase in the density ratio of the continuous fluid to the dispersed fluid caused a change in the MI profile and was a strong function of *Re*. For example, no significant change was observed when this ratio was 1, until *Re* = 144, after which, there was a sudden change with a nonlinear increase until *Re* ≤ 300. The authors attributed this to the different types of flows, as discussed in Section 2.1.

The simple design of Category 2 geometries showed good MI for low *Re*. An example is the study of Ansari et al. [69], who observed axial variation in MI as the *Re* varied between 1 ≤ *Re* ≤ 80. A comparison between Category 1 mixers (TJM) and Category 2 mixers (VJTM) showed that for lower Reynolds number (*Re* ≤ 1), there was no significant change in mixing indices between VTJM and TJM, but the MI showed a significant variation with an increase in *Re*. Similar studies by Khaydarov et al. [70] for Category 2 micromixers showed that for *Re* = 2.6, the MI had a linearly increasing profile along with the length of the microchannel (0 ≤ MI ≤ 0.2) while for *Re* = 259, the MI had a non-linearly increasing profile (0 ≤ MI ≤ 0.85) with the length of the microchannel (L ≤ 21 mm). The non-linear relationship was a linearly increasing profile until L ≤ 11 mm, then an exponentially increasing profile until 11 mm ≤ L ≤ 18 mm and a constant linear profile until 4 ≤ L ≤ 21 mm. The above relationship is a result of the application of vortex flow in a passive micromixer.

The design of Category 3 mixers involved an optimization of geometric shape, length, and number of obstacles in a micromixer and operating parameters like fluid velocities/flow rates. For instance, Borgohain et al. [71] numerically studied the effects of operating parameters on the mixing of a water–ethanol mixture in a Category 3 micromixer. The authors in their design of obstacles observed channeling phenomena at certain flow rates where the fluids took the shortest path, causing a lower amount of mixing. Due to this, the authors observed the parabolic relationship of MI with variation in *Re*. An important observation was the presence of a critical *Re* and *Pe*, at which the MI increased for their chosen *Re* range (0.1 ≤ *Re* ≤ 100). The MI was in the range (0.75 < MI < 0.88) after critical *Re*. While performing a sensitivity analysis of the effect of length and the number of obstacles on MI, the authors concluded that MI values were directly proportional to the number of obstacles (maximum of 10 obstacles) for all *Re* considered in their study. The authors have attributed this relationship to the increase in residence time due to increases in both length and number of obstacles. Several important findings included the dependence of MI on a critical number of obstacles, which provided maximum and cost-efficient mixing. Silva Jr et al. [72] found that obstacles were able to provide mixing at *Re* = 10 and increases in *Re* (10 < *Re* < 100) had no effect when a UCCFM micromixer was used. As per the authors, this version of the micromixer was suitable for low *Re*. The mixing index in the oil–ethanol system decreased until a minimum reached at *Re* = 10, with similar results observed for the three tested geometries. Further, the shape of the obstacles also played an important role in mixing depending on the type of fluid. For higher flow rates or highly viscous fluids, circular obstacles were used that caused reductions in the flow cross-section, as well as in velocity, inducing a split and recombination mechanism of the flow. Zhang et al. [35] observed in Category 3 micromixers (Koch Fractal Baffle Micromixers) that secondary flow patterns (high intensity chaotic advection) produced by micromixers working on fractal principles (SKFBM shown in Figure 3B) showed better performance in terms of mixing indices (0.8 < MI < 0.975) as compared with those producing primary flow patterns or low intensity of chaotic advection (PKFBM) while varying the Reynolds number in the range considered (0.1 ≤ *Re* ≤ 100). The high intensity of chaotic advection was obtained by having higher numbers of baffles/obstacles and the angles of baffles being placed than the PKFBM. The authors have attributed this to the complexity of the geometry of that caused flow variations.

Similar simulation studies using a different fractal micromixer, the Minkowski Fractal Micromixer, were carried out by Chen and Chen [74]. While the design of this micromixer was different than Zhang et al. [35], the conclusions drawn were similar in that secondary flow caused higher MI. The authors for the same *Re* range could, however, reach an MI value of 0.8 as compared with 0.98% by Zhang et al. [35]. The effects of the geometric parameters like the height of the baffles/obstacle and the number of obstacles were investigated. Dundi et al. [100] carried out investigations on the impact of swirl on the mixing efficiency of a T-mixer at different Reynolds numbers. The authors found that the specific structure of a swirl or vortex (denoted by dimensionless quantity termed as swirl number by authors) gave good mixing efficiencies for lower *Re* till *Re* = 106. For higher *Re* (*Re* > 250), chaotic mixing in the engulfment regime gave higher mixing, which was 3 to 5 times higher than those obtained for *Re* = 106. Farahinia and Zhang [75] found that the shape of the obstacles enhanced the mixing and improved efficiencies by 4 times, compared with simple micromixers. The increase in efficiency was found to be ~97% due to enhanced convective flux, and ~3% due to diffusion. The authors found that rectangular and diagonal obstacles and furrows inside the microchannel enhanced the convective flux, improving the mixing efficiency index and reducing the mixing length, leading to superior micro-T-mixer geometries. Okuducu and Aral [41] studied the effect of *Re* and the injection modes of fluids by splitting the inlets horizontally or vertically. While high MI values (~0.9) were found for low *Re*, the reverse was true for higher *Re*. The authors attributed this to the residence time (which ranges from high values for low *Re* and lower values for high *Re*) and the mixing mechanisms (diffusion for low *Re* and advection for high *Re*). The authors have tried different ways to achieve mixing by splitting the inlet vertically or horizontally.

Relatively good MI values have been noted in Category 4 mixers as compared with Categories 2 and 3, as reported in the literature. Tran-Minh et al. [61] found a high MI of 0.85 for their SAR mixers with low pressure drops as compared with other micromixers of similar designs. Further, Ruijin et al. [40] found that Baker mixers provided MI values of around 0.9 and were best suited for low *Re* applications while SARs provided MI values of around 0.9 and were well suited for high *Re* applications. In investigations by Hou et al. [33], the range of *Re* was between 0.1 and 150. For lower *Re* numbers, low mixing efficiencies were found while for *Re* = 50, a mixing efficiency of around 94% was reached. Okuducu and Aral [41] found that the mixing index improved by 8.7 times for novel mixtures as compared with classical TJMs. One of the most significant variations was found not only in geometry but also flow arrangement. The authors created a horizontal or a vertical split for inlets and have provision for alternate injection. These injection strategies helped to change flow patterns and improve mixing indices over and above improvement due to obstructions. The *Re* range was kept from 0.1 to 240 (*Re* = 0.1, 0.5, 1, 5, 10, 20, 40, 80, 160, 240. Mixing efficiencies were found to be more than 80%.

The pressure drops for the above mixers will be described in the next section.

## 4. Pressure Drop in of Passive Micromixers

As discussed in Section 2, although passive mixers were characterized by simple geometries, the presence of obstructions/meanders/complex geometrical modifications increases the pressure drop passes through the passive micromixer. Further, pressure drop values, since they influence overall device performance, are primarily a strong function of Reynolds numbers and, in turn, a strong function of operating parameters like the flow rate of fluids’ fluid properties and geometrical parameters like the hydraulic diameter. It is important to note that the pressure drop in passive micromixers is typically lower compared with traditional macro-scale mixers due to the small channel dimensions and short flow lengths. The aim of having any passive mixer is to have a maximum mixing without exorbitant increases in pressure drop. Various analytical, computational, and experimental methods have been employed to estimate or measure the pressure drop in passive micromixers, depending on the complexity of the mixer design and the desired accuracy.

In this section, we discuss the various factors that affect pressure drop and the research works carried out by authors on different factors on which the pressure drop depends.

### 4.1. Analytical and Empirical Expressions for Pressure Drop in Passive Micromixers

Table 5 shows the different analytical/empirical expressions for pressure drop reported by different researchers for passive micromixers.

Due to a well-mixed system of two fluids under consideration, the analytical expressions for the passive mixers are similar to those of flow through pipes while empirical expressions from experimental data have been developed by several authors. The analytical expressions include the Darcy Weisbach Equation and Hagen Poisuelle equations with modifications in friction factor expressions due to mixing elements.

Most of the time, in the passive micromixers, the fluid flow is at every Reynolds number. Hence, the fluid flow is considered to be laminar during the flow. The Hagen Poiseuille equation is an analytical expression for the determination of the pressure drop of a steady state incompressible in a cylindrical geometry with a constant cross-section. Babu et al. [90] performed experimental measurements and numerical simulations for an *Re* range (100 < *Re* < 400) and found an empirical correlation for pressure drop in TJM and YJM. Here, ξ the effective friction factor was found as 5.2 from the regressed experimental data. The value of f_D_ was found to be 0.23 for the *Re* range, which the authors found comparable to the *Re* range.

Khaydarov et al. [70] numerically derived pressure drop in their Category 3 micromixers as a function of the Reynolds number. Hosseini and Rahimi [76] used an overall friction factor which combines the pressure drop using Darcy friction and pressure drop using local loss.

### 4.2. Effect of Geometric and Operating Parameters on Pressure Drop in Passive Micromixers

As briefly discussed in previous sections, geometric and operating conditions play a major role in defining the performance of a micromixer. We now revisit in detail the pressure drop aspects in this section. The influence of operating conditions like flow rate include flow rate variations that affect the flow behavior and pressure drop with a direct proportionality. Hence, the properties of the fluids being mixed, such as viscosity, density, and surface tension, can impact the pressure drop in a micromixer.

Geometric conditions include the type of micromixer inlet and outlet that affects the flow distribution and velocity profiles, which in turn influence the pressure drop. Non-uniform flow distributions or high-velocity gradients can result in uneven pressure drops along the micromixer length. Other geometric parameters include the design of the inlet and outlet ports of the micromixer, the design of inlet and outlet, whether the inlet is single or multiple or combined, and the design and dimensions of the microchannel itself, like the width, length, and depth of the channel, and the type of the channel (e.g., straight channels, serpentine channels, or complex microstructures), generally represented by aspect ratio (AR) (the ratio of the height of the channel to the width of the channel). AR has an inverse relationship with (∆P) due to increased flow resistance. Micromixers consists of constrictions, which narrow the channel width, obstacles, or microstructures within the micromixer which can increase vortex formation, flow recirculation, or flow resistance, all of which increase the pressure drop. This depends on the shape, size, and distribution of these structures in the channel. These structures include obstacles, baffles, grooves, or other geometrical features that enhance mixing, and play a role in improving the mixing efficiency, but induces additional flow resistance causing an increase in ∆P. Another factor impacting ∆P is the overall topology or layout of microchannels. Typical topologies include parallel channels, meandering channels, or interconnected networks. Finally, the properties of the micromixer materials, such as their surface roughness, can impact the pressure drop. With a direct proportionality, rougher surfaces can lead to increased frictional losses and, consequently, higher pressure drops. The dimensional tolerances and surface roughness of the micromixer components can impact the pressure drop. Variations or imperfections in the fabricated features, such as channel width or surface roughness, can introduce additional flow resistance and affect the pressure drop characteristics.

For certain micromixers (for example, TJMRWP, Borgohain et al. [71]), a linear relationship of pressure drop with *Re* was observed for *Re* in the range (0.1 ≤ *Re* ≤ 100). Capillary numbers of both fluids (water and ethanol) were less than 5.63 × 10^−3^. Further, critical *Re* numbers, after which bypassing was observed, were also reported by the authors. Further, Borgohain et al. [71] observed that with an increase in the length of the obstacles between 0.1 μm < l < 0.225 μm, the pressure drop increased linearly between 80 Pa < ΔP_TJMRWP_ < 111 Pa due to an increase in the contact surface. Further, an increase in the number of rectangular winglet pair obstacles between 0 ≤ *N_m_* ≤ 10 also had a linear increase in the pressure drop (51 Pa < ΔP_TJMRWP_ < 171 Pa) and hence the pressure drop is a function of the number of modules of obstacles within the system. Researchers investigating with micromixers of Category 2 (for example, Khaydarov et al. [70]) have observed a parabolic relationship in pressure drop (for *Ca* < 3.61 × 10^−3^ and 0 ≤ *Re* ≤ 260) and a critical *Re* when this breakeven point occurs. Pressure drop per meander length was found to increase linearly for lower Reynolds numbers from *Re* = 0.3 to *Re* = 26. Some researchers investigating micromixers in Category 3 [65] have found that after *Re* = 60, the pressure drop increases exponentially. For example, for *Re* = 100, the pressure drop becomes double (Δ*P* = 15,000 Pa) as that of *Re* = 60. Further, the increases in mixing efficiencies were not found to be sufficient to accommodate the pressure drop. Lobasov and Minakov [27] found some interesting phenomena during their numerical studies of how the fluid properties (like the density and viscosity ratio) and temperature difference between two fluids affected the mixing and pressure drop in TJM with a water–dye mixture for Capillary numbers Ca < 0.016. Interestingly, the pressure drop was found to decrease with an increase in the density ratio while an increase with an increase in the viscosity ratio of the continuous fluid was observed at any constant Reynolds number. Further, it was found that the pressure drop depended on temperature difference between continuous and dispersed phases. The maximum pressure drop was observed when temperature difference between the continuous and dispersed fluid was −10 °C. Chen and Chen [74] during their analysis of fractal micromixers with obstacles found that the ∆P depended on the height of the obstacle and the rate of increase in ∆P was non-linear after *Re* = 10. Hosseini and Rahimi [76] compared their four different micromixer designs and found that the Cylindrical Barrier Micromixer (CBM) exhibited the highest pressure drop, 1.8 to 2.4 times higher than the plain microchannel, due to the formation of more vortices. The lowest pressure drop was shown by their Semi Conical Barrier Micromixer design (SCoM) due to the absence of fluid rotation. Okuducu and Aral [41] carried out studies in Category 1 micromixers (T-shaped micromixer (CT)). The pressure drop remained below 1 kPa until a Reynolds number (*Re*) of 40, while the maximum pressure drop, exceeding 15 kPa, was observed in the engulfment flow regime, while for low *Re* values of 20 and 40, the pressure drops were as low as 2.07 and 4.72 kPa, respectively. One of their micromixer designs improved energy efficiency with lower pressure drops compared with the classical T-shaped micromixer and other micromixers. Niu et al. [32] had trapezoidal structures in their micromixer which caused expanding vortices causing a change in Dean vortices across the curvature of channel. These secondary flow structures caused change in mixing efficiency. However, an increase in pressure drop was observed due to an increase in overall flow area. Pressure drops were found to be lower than that in equivalent staggered baffles. The curved staggered baffles applied to T-junctions and serpentine-curved channels were found to be efficient as compared with T-junctions and serpentine channels without curved staggered baffles.

## 5. Discussion

This section focuses on the analysis of pressure drop and MI data to find the following: (1) A comparison of the designs of micromixers of different categories was made based on the measured pressure drop to one obtained if the micromixer were a microchannel; (2) a determination of the operating region for efficient operation of micromixers based on optimum MI and pressure drop was conducted; (3) a classification of MIs based on different regimes, namely diffusion, convection, and chaotic motion, was made. First, the percentage increase in the pressure drop in magnitude in a micromixer, as compared with that in a microchannel, and the corresponding increase in MI needs to be understood. Table 6 shows a comparison of the pressure drops in micromixers for different categories. The comparison is made as follows: The reported data of pressure drop (either predicted by numerical simulations or measured using experimental measurements) of the selected micromixers are collated. The Hagen–Poiseuille equation is applied to calculate the pressure drop if the micromixer were a microchannel for laminar flow through the microchannel. The microchannel was assumed to have a hydraulic diameter without mixing elements. The % increase in pressure drop due to micromixing elements for each of the reported data is shown in Table 6. Most of the data shown in the Table 6 are at *Re* (*Re* = 50 or near to 50) so that a good comparison between all the categories can be obtained. Two different *Re* values, namely *Re* = 40 and *Re* = 100, have been also been shown to report a wider range of *Re* and the unavailability of data by the authors. However, the objective is to compare different category micromixers for the same *Re*. Clearly, the pressure drop increases with an increasing degree of complexity with Category 1 having the lowest percentage increase and other categories having higher increases in pressure drop, except two micromixer designs by Niu et al., 2022 [32], which have a pressure drop higher than 1000% or an order of magnitude higher than if the micromixer was a straight channel. Though, such designs might provide an excellent mixing index, such designs should be avoided since they increase both the energy consumption and the cost. From the chosen micromixers, the micromixer of Mahmud et al., 2021, (Category 2) and Yuan et al. [87] (Category 4) prove to be very efficient micromixers that have a lower pressure drop and a high mixing index. This also suggests that through adding obstructions, Category 3 increases the mixing characteristics in the system but also increases the pressure drops drastically. Category 1 micromixers have a low pressure drop so the mixing index is very low. Hence, flow optimizations need to be performed to obtain a higher mixing index but a lower pressure drop, which, from the selected micromixers, seems to be in one of the Category 4 passive micromixers.

Once the percentage increase in pressure drop for different micromixer designs, compared with microchannels, is understood, the operating region for any micromixer needs to be defined. This region would serve as a preliminary estimate for the selection of a micromixer design before an optimum evaluation of pressure drop vs MI is conducted.

**Figure 4:**
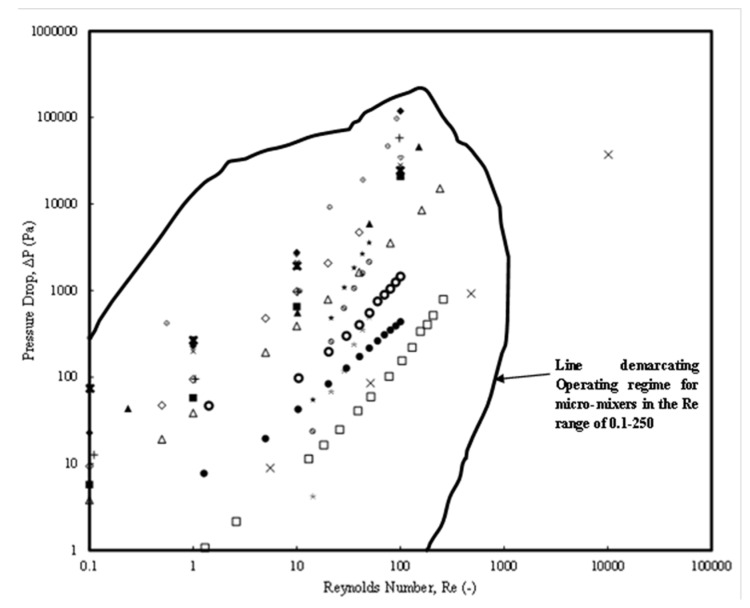
Pressure drop variation with *Re* for different categories of micromixers 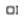 Khaydarov et al. [70] (LTFMSM); 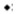 Chen and Chen [74] (SMFOM); 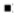 Chen and Chen [74] (PMFOM) 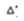 Okuducu and Aral [41] (TLM); 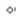 Okuducu and Aral [41] (CSCRM); 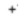 Lotfiani and Razazadeh [81] (YJM); 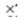 Lotfiani and Razazadeh [81] (TLM); 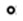 Mahmud et al. [83] (HCM); 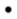 Mahmud et al. [83] (YUM); 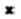 Tripathi et al. [85] (SpiM); 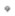 Tripathi et al. [85] (RsPM); 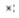 Tripathi et al. [85] (SESpM); 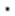 Niu et al. [32] (ESBM); 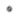 Niu et al. [32] (CSBM); 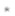 Niu et al. [32] (SBM); 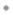 Yuan et al. [87] (ASZMMM); 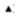 Hou et al. [33] (TSFOM).

Figure 4 shows the pressure drop variation with a Reynolds number (in the range 0.1 < *Re* < 250). Data from the prominent and current literature have been collated and presented. Most of the micromixers showed a linear variation with a maximum pressure drop of 100 kPa for a Category 3 micromixer (SMFOM) (Chen and Chen [74]) (for *Re* = 100) while a minimum pressure drop of around 1 kPa is observed for same *Re* for the Category 2 micromixer (Khaydarov et al. [70]). An operating window for the micromixers has been defined by the region within which any of the micromixers can be selected depending on the requirement. Further, new micromixers need to be designed such that they lie within this region for the operable *Re* range. This would help design engineers to select existing micromixers or design new micromixers. However, this analysis needs to be verified with the mixing index values for each of the pressure drops. This will be discussed in a later part of the discussion.

The pressure drops included in Figure 4 consist of the ones from experimental investigations in the range (1 Pa ≤ Δ*P* ≤ 100 kPa) with *Re* ranges (0.1 ≤ *Re* < 1000) while those from numerical investigations were in the range (1 Pa ≤ Δ*P* ≤ 100 kPa) and the Reynolds number range (0.1 ≤ *Re* < 250). An important observation is that the micromixer design of Yuan et al. [87] (Category 4) can also be selected, which has a corresponding pressure drop of around 19,000 Pa for a *Re* = 50 and MI values of around 1.

In Table 6, MI values have been presented from the literature data available in the published literature from numerical investigations. However, the number of grid elements or grid resolutions across the spatial domain is crucial for a proper mixing prediction (K Sommer [98]). The grid elements selected for presenting the comparison of MI obtained by different authors with different grid elements were first tested based on the number of grid elements. Table 7 also shows that the grid resolutions for the Category 3 and 4 micromixers are higher than those of the Category 2, except for Niu et al. [32], in which the smallest grid resolution was much coarser than the others.

Due to the complex structure and nonuniform grid for the geometries of all the authors, the analyses might present differences in the cell volumes, as shown in Table 7.

**Figure 5:**
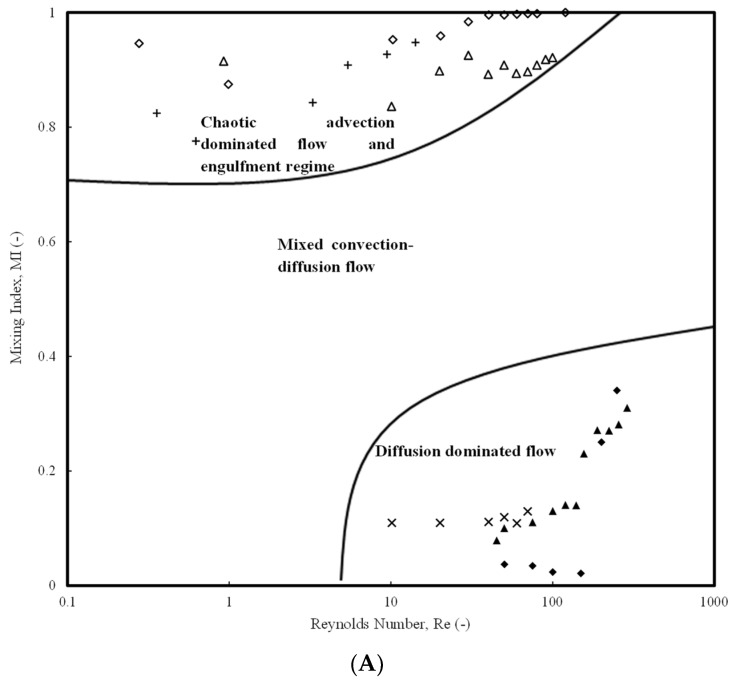

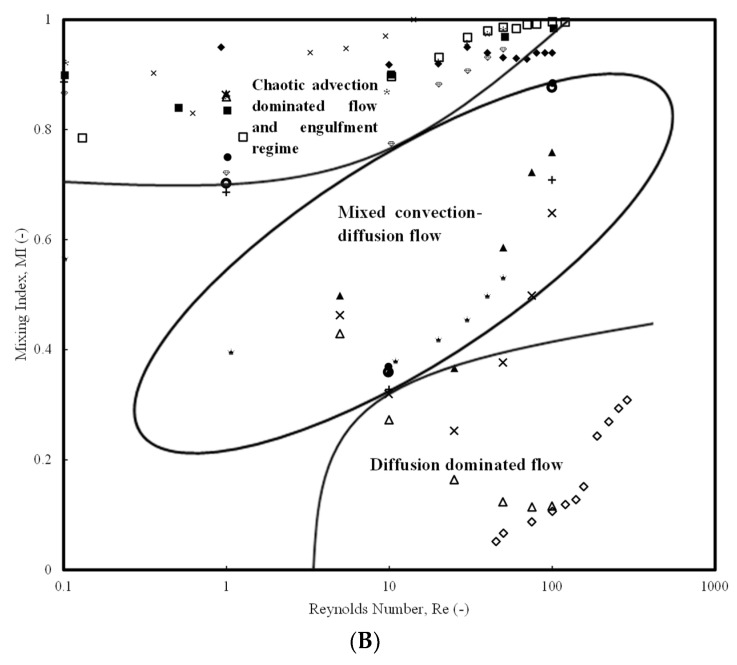
Mixing index of micromixers available in the literature: (**A**) Experimental Investigations, Ansari et al. [69] (TJM); Raza et al. [25] (TLCCM); Yuan et al. [87] (ASZMMM); Silva Jr, et al. [72] (TJM); Viktorov et al. [103] (TJM); Cortes-Quiro et al. [104] (HCM); (**B**) Numerical Studies. 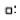 Amar et al. [77] (TJM); 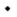 Mahmud et al. [83] (YUM); 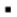 Tripathi et al. [84] (YJM); 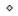 Tripathi et al. [84] (SM); 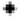 Tripathi et al. [84] (SpiM); 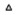 Tripathi et al. [85] (SpiM); 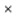 Tripathi et al. [85] (RsPM); 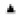 Tripathi et al. [85] (SESpM); 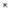 Yuan et al. [87] (ASZMMM); 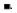 Hou et al. [33] (TSFOM); 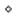 Okuducu and Aral [78] (TJM); 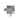 Niu et al. [32] (ESBM); 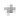 Niu et al. [32] (CSBM); 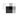
Niu et al. [32] (SBM).

Figure 5A shows the details of MI based on experimental investigations. From the figure, it can be observed that the passive micromixers are divided into three major areas: (1) MI range (0 < MI < 0.4) and *Re* range (6 < *Re* < 500), which is predominantly a diffusion-dominated flow in the symmetric or (2) MI range (0.7 < MI < 1) and *Re* range (0.1 < *Re* < 500), which is dominated by chaotic advection-dominated flow or stochastic flow in the engulfment regime. For Category 1 micromixers, the MI values were found to be very low. For example, the investigations of Okuducu and Aral [71] found that TJM showed lower MI values in the *Re* range (50 < *Re* < 300) (MI < 0.4) while the Category 3 mixer (TJMRWP) by Borgohain et al. [60] showed MI values (0.6 < MI < 0.99) for the *Re* range (1 < *Re* < 10). Similarly, the Category 4 micromixer designed by Amar et al. [77] (TLCCM) showed MI values ranging from 0.7 to 1 in the *Re* range 0.1 < *Re* < 100. One can also observe that for low Reynolds numbers, 0.1 < *Re* < 1, this situation might be valid only with micromixers in Categories 3 and 4 for those that have high pressure drops but better mixing charastersitics. However, the mixing behavior is different for low *Re* flows. For such flows, the MI values decrease for Re in the range (0.1 < *Re* < 1) and undergo a minima at a certain *Re* (example, *Re* = 10) and then increase. The minima occurs at 0.7 in these cases. For example, for the Category 4 micromixer designed by Amar et al. [69], (TLCCM) the MI values decreased from 0.1 < *Re* < 1, encountered a minima between 1 < *Re* < 10, and increased till *Re* ≈ 100 with MI values of 1 at *Re* ≈ 100. Other micromixers of Category 4 also observed MI values undergoing a minima for a slightly higher *Re* range (30 < *Re* < 100). For example, a Category 4 micromixer (HCM) designed by Mahmud et al. [83] also found that MI (0.85 < MI < 0.95) values encountered a minima with increases in *Re*. An increasing trend of MI values (0.65 < MI < 0.8) was observed by Yuan et al. [87] for a Category 4 micromixer (ASZMMM) for the *Re* range (1 < *Re* < 100) which did not undergo a minima.

**Figure 6:**
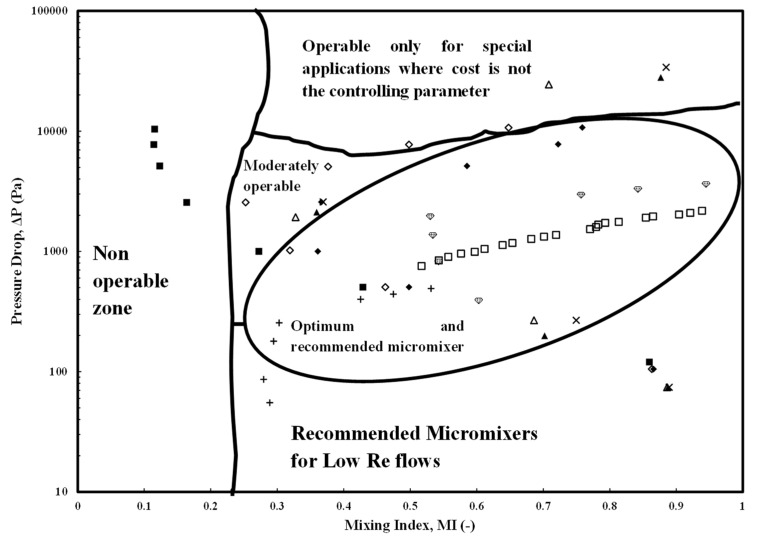
Map for selection of micromixer based on pressure drop v/s mixing index for a *Re* range of 1 ≤ *Re* ≤ 100. 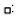 Talebjedi et al. [80] (TJM); 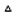 Tripathi et al. [84] (YJM); 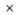 Tripathi et al. [84] (SM); 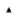 Tripathi et al. [84] (SpiM); 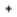 Tripathi et al. [85] (SpiM); 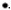 Tripathi et al. [85] (RsPM) 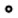 Tripathi et al. [85] (SESpM); 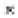 Yuan et al. [87] (ASZMMM); 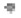 Niu et al. [32] (ESBM); 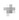 Niu et al. [32] (CSBM); 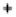 Niu et al. [32] (SBM).

While Table 4 would help a design engineer to select the category of the micromixer design (Category 2, 3, or 4) for a particular Reynolds number, a comprehensive analysis of MI values for corresponding pressure drops would give a design engineer the means of selecting a micromixer design based on the needed MI value and its corresponding pressure drop. Figure 6 presents such an analysis where different categories of design show the variation of pressure drop versus the mixing index for the Reynolds number range (1 ≤ *Re* ≤ 100) for different categories of micromixers. Since the Category 2 micromixer by Tripathi et al. [78] gives 90% MI with a pressure drop of 100 Pa, a design engineer can select this micromixer as it has an optimized pressure drop and MI values.

## 6. Empirical Expression for Friction Factor

From the literature review, it was observed that a single correlation for pressure drop in the case of micromixers is not available for a wide range of *Re*. Hence, all the publicly available pressure drop data from the recent available experimental data from the literature (for last 5 years) have been collected for an *Re* range (0.1 ≤ *Re* ≤ 100) and the following correlation for Fanning’s friction factor (similar to the approach of Ganguli and Pandit [105]) has been presented.

(1) $$f=40.95{Re}^{-0.95}$$

For miscible systems, the Chilton–Colburn analogy can be used for heat and mass transfer correlations. Hence, to find the overall mass transfer coefficient (K_L_), the below equations can be used

(2) $$\frac{f}{2}=St{Sc}^{0.66}\;\mathrm{where}\;\mathrm{St}\;\mathrm{represents}\;\mathrm{the}\;\mathrm{Stanton}\;\mathrm{number},\;St=\frac{Sh}{ReSc}$$

To find the heat transfer coefficient, the below equation can be used,

(3) $$\frac{f}{2}=St{Pr}^{0.66}\;\mathrm{where}\;\mathrm{St}\;\mathrm{represents}\;\mathrm{the}\;\mathrm{Stanton}\;\mathrm{number},\;St=\frac{Nu}{RePr}$$

The friction factor f in Equations (2) and (3) are calculated using Equation (1).

**Figure 7:**
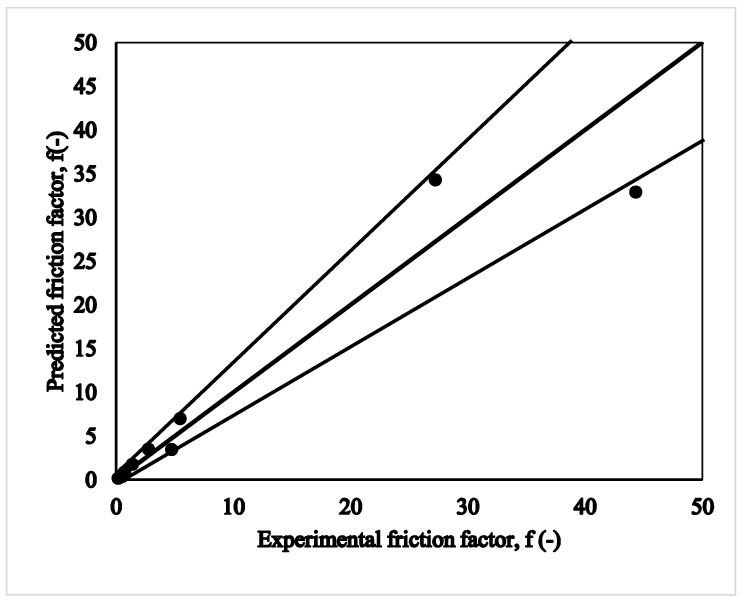
Parity plot of friction factor as per experiments and predicted by Equation (1).

Figure 7 shows the parity plot of the predicted data for the empirical correlation given by the present work in the *Re* range (1 ≤ *Re* < 300). It can be observed that the deviations are restricted to ±25%.

## 7. Conclusions

The following conclusions can be drawn from the studies:

1. The categorization and appropriate abbreviation of micromixers introduced in the present work will contribute to the understanding flow, transport, and performance characteristics of micromixers. The categorization has been based on the size, shape, layout, whether or not it is curved or having obstructions, or a combination of shapes and complex shapes, inlet and outlet configuration, and the flow rate.
2. Although MI has largely been considered as having a linear relationship with flow rate, at a certain *Re*, Category 1 mixers might undergo an S-shaped structure or non-uniform profile, inducing rapid mixing and increasing the MI values.
3. The Peclet number is an important parameter for understanding the mixing. Diffusion coefficient influences mixing speed, with higher values promoting rapid mixing. Concentration disparities and mixing ratios influence MI while the mixing protocol, including injection sequence and timing, and the duration of the mixing process also affect mixing efficiency.
4. Flow regimes over a wide range of Reynolds numbers depend on two aspects, the category of the micromixers and the Reynolds number.
5. In Categories 1 and 2, the MI values are restricted to 0.1 < MI < 0.6 for *Re* range, 40 < *Re* < 300, while certain designs in Category 2 like helical coil give high MI (~upto 0.8) values due to the dean vortices and lower pressure drops. Categories 3 and 4 give MI values between 0.7 and 1 for a wide range of *Re* from 0.1 to 100. An interesting trend in Mi is observed with MI undergoing minima for lower *Re* in the range considered and then increasing with increases in *Re*.
6. The MI can be distinctly divided into three zones with low MI values from 0.1 to 0.4 for Category 1 micromixers where the mixing is diffusion dominated, Category 2 and 3 mixed convection diffusion in the MI range 0.4 to 0.7, and Category 2 (helical mixers), but mostly Category 3 and 4, which have chaotic or stochastic motion for the MI range 0.7 to 1.
7. A comprehensive representation of pressure drop versus MI is presented for the optimum operable range for micromixers. This is expected to help the design engineer to select an optimum design of micromixer for a smooth and economically viable operation. The figure also recommends the non-operable region, the operable region for low *Re* flows, and the operable region for special flows.
8. A comparison of pressure drop in micromixers with those of microchannels shows some micromixers having an increase in pressure drop by two to three orders of magnitude. Hence, the design and selection of micromixer should be made with caution, preferably within the design limits of the optimum range proposed in the present work.
9. A correlation for friction factors based on the pressure drop from the literature data has been developed which will help in also understanding the heat and mass transfer characteristics.

## Figures and Tables

**Table 1:** Various Types of Geometries used as Micromixers.

Reference	Geometry	Geometry Abbreviation	Category
Aubin et al. [42]	Staggered Herringbone Micromixer	SHM	2
Jin et al. [29]	Swirl Micromixer	SwM	2
Adeosun and Lawal [36]	Multilaminated/Elongational Flow Micromixer	MEFM-4	2
	T-Junction Micromixer	TJM	1
Malecha et al. [43]	I-Shaped Serpentine Micromixer	ISSM	2
Balan et al. [44]	Y-Junction Micromixer	YJM	1
Du et al. [45]	Slanted Groove Micromixer	SGM	2
Ait Mouheb et al. [46]	Cross Flow Junction Micromixer	CFJM	1
Rudyak et al. [47]	T-Junction Micromixer with Rectangular Inserts	RITJM	3
Fang et al. [48]	Overlapping Micromixer	OM	3
Chen et al. [49]	Crosswise Ridge Micromixer	CRM	3
Dreher et al. [50]	T-Junction Micromixers with Vertical Rectangular Cross Sections	TJMVRCS	3
Düchs et al. [51]	Berger Ball Micromixer	BBM	4
Tseng et al. [52]	Diamond Obstructed Y-Junction Micromixer	DOYJM	3
Fang et al. [53]	Periodically Obstructed Micromixer	POM	3
Nieves-Remacha et al. [39]	Corning Advanced Flow Reactor (Immiscible system)	CAFR	4
Fischer and Kockmann [54]	Tangential T-Junction Micromixer	TaTJM	4
	Twisted Y-Junction Micromixer	TYJM	4
	Inverted Twisted Y-Junction Micromixer	ITYJM	2
Roudgar et al. [55]	Horizontally Splited T-Junction Micromixer	HSTJM	2
	Vertically Splited T-Junction Micromixer	VSTJM	2
Leung and Ren [56]	Obstructed and Width Constricted Micromixer	OWCM	3
Wang et al. [57]	Tree-Shaped Micromixer	TSM	4
Plouffe et al. [58]	Venturi Micromixer (Immiscible system)	VM	4
	Cylindrical Serpentine Micromixer (Immiscible System)	CSM	2
Cheri et al. [59]	Hexagonal Chamber Micromixer	HeCM	4
	Round Corner Rectangular Chamber Micromixer	RCRCM	4
Su et al. [60]	Zigzag T-Junction Micromixer	ZTJM	2
Tran-Minh et al. [61]	Elliptical Obstructed Splitting and Recombination Micromixer	EOSARM	4
Parsa et al. [62]	Sinusoidal Micromixers	SiM	2
Rudyak and Minakov [34]	T-Type Micromixer with Rectangular Obstacles	ROTJM	3
Picardo and Pushpavanam [63]	Curved Micromixer	CM	2
Chandra et al. [64]	Enhanced Micromixer	EM	3
Chen et al. [65]	Square wave Serpentine Micromixer	SSM	2
	Multi wave Serpentine Micromixer	MSM	2
	Zigzag Serpentine Micromixer	ZSM	2
Luo et al. [28]	Helical Coiled Tube Micromixer	HCTM	2
Plouffe et al. [38]	SZ Micromixer (Immiscible system)	SZM	4
	TG Micromixer (Immiscible system)	TGM	4
	Sickle Micromixer (Immiscible system)	SicM	4
	Spade Micromixer (Immiscible system)	SpM	4
Huang et al. [66]	Longitudinal Modules Vortex Micromixer	LMVM	3
Ortega-Casanova [67]	Obstructed Micromixer with Square Cylinder	OMSC	3
Akbarzadeh et al. [30]	Sinusoidal Wavy Channel Micromixer	SWCM	2
	Trapezoidal Wavy Channel Micromixer	TWCM	2
	Triangular Wavy Channel Micromixer	TrWCM	2
Ruijin et al. [40]	Baker Micromixer	BM	4
	Smale Micromixer	SmM	4
	Helical Micromixer	HM	4
Xiong et al. [68]	Co-Axial Micromixer	CAM	2
Ansari et al. [69]	Vortex T-Junction Micromixer	VTJM	2
Khaydarov et al. [70]	LTF-MS Micromixer	LTFMSM	2
Borgohain et al. [71]	T-Junction Micromixer with Rectangular Winglet Pair	RWPTJM	3
Silva Jr et al. [72]	Uniform Circular Obstacles and Cross Flow	UCCFM	3
Zhang et al. [35]	Primary Koch Fractal Baffle Micromixer	PKFBM	3
	Secondary Koch Fractal Baffle Micromixer	SKFBM	3
Cheng et al. [73]	Single Countercurrent Flow Micromixer	SCFM	2
Li et al. [13]	Pore Array intensified Tube-in-tube Micromixer	PAITTM	4
Chen and Chen [74]	Primary Minkowski Fractal Obstacle Micromixer	PMFOM	3
	Secondary Minkowski Fractal Obstacle Micromixer	SMFOM	3
Rahmannezhad and Mirbozorgi [31]	Circular Obstructed Grooved Micromixer	COGM	3
	Diamond Obstructed Grooved Micromixer	DOGM	3
	Square Obstructed Grooved Micromixer	SOGM	3
Farahinia and Zhang [75]	Rectangular Obstacle T-Junction Micromixer	ROTJM	3
	Diagonally Obstacle T-Junction Micromixer	DOTJM	3
	Diagonally Primary Minkowski Fractal Obstacle Micromixer	DPMFOM	3
Hosseini and Rahimi [76]	Cylindrical Barrier Micromixer	CBM	3
	Semi Cylindrical Barrier Micromixer	SCBM	3
	Conical Barrier Micromixer	CoBM	3
	Semi Conical Barrier Micromixer	SCoBM	3
Okuducu and Aral [41]	Convex Semi-Circular Ridge Micromixer	CSCRM	2
Silv Jr. et al. [72]	Circular Obstacle Cross Flow Micromixer	COCFM	3
Amar et al. [77]	Two Layer Crossing Channel Micromixer	TLCCM	4
Hou et al. [33]	T Symmetry Fractal obstacle micromixer	TSFOM	3
	T Asymmetry Fractal obstacle micromixer	TASFOM	3
	Semicircle T Symmetry Fractal obstacle micromixer	STSFOM	3
	Semicircle T Asymmetry Fractal obstacle micromixer	STASFOM	3
Okuducu and Aral [78]	Circular-Shaped Fluid Overlapping Micromixer	CSFOM	4
Zhang et al. [37]	Random Micromixers	RM	4
Feng et al. [79]	Flexible Rubik’s Cube Module Micromixer	FRCMM	4
Talebjedi et al. [80]	Rigid Baffles T-Junction Micromixer	RBTJM	3
	Deformable Baffles T Junction Micromixer	DBTJM	3
Lotfiani and Rezazadeh [81]	Two Layer Micromixer	TLM	4
Wang et al. [82]	Diamond Obstacle Tesla Micromixer	DOTM	3
Mahmud et al. [83]	YU Micromixer	YUM	4
	HC Micromixer	HCM	4
Tripathi et al. [84]	Spiral Micromixer	SpiM	2
Tripathi et al. [85]	Sharp Edged Spiral Micromixer	SESpM	2
	Rectangular Spiral Micromixer	RSpM	2
Valeh-e-Sheyda and Yarmohammad [86]	Trapezoidal T-Junction Micromixer	TrTJM	2
	Concentric Micromixer	CoM	2
	Caterpillar Micromixer	CaM	2
Yuan et al. [87]	ASZMM Micromixer	ASZMMM	3
Bahrami and Bayareh [26]	Sinusoidal Walls Spiral Micromixer	SWSM	2
Niu et al. [32]	Staggered Baffles Micromixer	SBM	3
	Cross Scale Staggered Baffles Micromixer	CSBM	3
	Equivalent Staggered Baffles Micromixer	ESBM	3
Shanbhag et al. [88]	X-Shaped Micromixer	XsM	2
Karthikeyan et al. [89]	Obstacle Serpentine Micromixer	OSM	3

**Table 2:** Recent Experimental Investigations in Micromixers.

Reference	Geometric Dimensions	Fluid Details	Reynolds Number, Peclet Number, and Capillar Number	Pressure Drop (ΔP) Details	Mixing Index (MI) Details
Parsa et al. [62]	0.06 cm < L < 1.4 cm; W = 200 mm; 100 μm < H < 322 μm	Methylene blue, water	*Re* < 50	ΔP < 30,000 Pa	0.2 < MI < 1
Chen et al. [65]	-	Blue ink, yellow ink	0.1 < *Re* < 200	ΔP < 50,000 Pa	0.3 < MI < 1
Babu et al. [90]	W = 0.003 m; L = 0.0016 m; H = 0.001 m	Water–Water (with certain concentration)	100 < *Re* < 400	10 Pa < ΔP < 10,000 Pa	0.175 < MI < 0.4
Ansari et al. [69]	W = 200 μm; H = 200 μm; L = 5 mm	Water, ethanol	1 ≤ *Re* ≤ 80	-	0.01 < MI < 0.45
Khaydarov et al. [70]	D = 1 mm; L = 21 mm	Colored ink, water	1.3 ≤ *Re* ≤ 259.3	1 Pa < ΔP < 789 Pa	0.33 ≤ MI ≤ 0.80
Hosseini and Rahimi [76]	D = 0.9 mm; L = 10 cm	Water, ARS, Aliquat, 1-octanol	47 ≤ *Re* ≤ 377	15 mbar < ΔP < 175 mbar	Performance ratio: 1 to 1.7
Mariotti et al. [91]	W = 1 mm; L = 45 mm	Methylene blue, ascorbic acid	*Re* < 700	-	-
Yuan et al. [87]	L > 4000 μm	Water, ethanol	0.1 ≤ *Re* ≤ 50	0.002 kPa < ΔP < 100 kPa	0.77 < MI < 0.99
Bahrami and Bayareh [26]	W = 200 μm; L > 70 mm	Water–Concentrated water	0 ≤ *Re* ≤ 100	0.1 kPa < ΔP < 40 kPa	0.15 ≤ MI ≤ 0.99

**Table 3:** Recent Numerical Studies in Micromixers.

Reference	Geometric Dimensions	Fluid Details	CFD Software Details	Mesh Details	Reynolds Number Range	Pressure Drop (ΔP) Details	Mixing Index (MI)/Segregation Index (SI) Details
Amar et al. [77]	*D_h_* = 350 μm	Water–dye	ANSYS Fluent 16.0	M1 = 222,917 M2 = 429,113 M3 = 643,203 M4 = 1,083,357	*Re* < 120; 150 ≤ *Pe* ≤ 13,840	ΔP < 90 kPa	0.1 < MI < 1
Mariotti et al. [91]	H = W = 1 mm; L = 45 mm	Methylene blue, ascorbic acid	ANSYS Fluent v.19	M = 4,700,000	*Re* < 700	-	-
Okuducu and Aral [78]	Inlet and exit W = 200 μm and diameter of 300 μm for circular part	Water and fluid with diffusivities 0.3, 1.5, and 3 nm^2^/s	OpenFOAM v5.0	M1 = 3,900,000 M2 = 2,450,000 M3 = 1,580,000 M4 = 1,050,000	0.1 ≤ *Re* ≤ 300; 280 ≤ *Pe* ≤ 82,000	100 ≤ ΔP ≤ 14,000	0.5 ≤ MI ≤ 0.92
Hou et al. [33]	Fractal shape with main channel W = 90 μm; L = 300 μm; other branches 45 and 22.5 μm width and 150 and 75 μm length	Water with concentration from 0 to 1 mol/lit	COMSOL Multiphysics 5.2a	M1 = 41,635 M2 = 79,089 M3 = 155,794 M4 = 338,441	0.1 ≤ *Re* ≤ 150	ΔP < 50,000 Pa	0.7 < MI < 1
Talebjedi et al. [80]	H = 300 μm; L ≤ 8000 μm; deformable baffle heights < 125 μm and width = 20 μm	Water–water with concentration from 0 to 1 mol/m^3^	COMSOL Multiphysics 5.4	-	*Re* < 60	ΔP < 3500 Pa	MI < 0.9
Lotfiani and Rezazadeh [81]	100 μm ≤ H ≤ 200 μm; 2800 μm ≤ L ≤ 20,000 μm	Water–water with concentration from 0–1 mol/m^3^	ANSYS Fluent 17.0	M1 = 107,078 M2 = 224,000 M3 = 233,600	1 < *Re* ≤ 100	10 Pa ≤ ΔP ≤ 100,000 Pa	MI ≤ 0.995
Wang et al. [82]	W = H = 100 μm; L = 7 mm	Water–water	COMSOL Multiphysics 5.5	M1 = 205,120 M2 = 353,085 M3 = 523,973 M4 = 710,479 M5 = 909,697	4 ≤ *Re* ≤ 200	198.4 ≤ ΔP < 536.02 Pa	0.728 ≤ MI ≤ 0.964
Mahmud et al. [83]	L = 7 mm; W = 400 μm	Water–water with diffusivity 1 nm^2^/s	ANSYS Fluent 15	200,000 < M < 1,000,000	0 ≤ *Re* ≤ 100	ΔP < 1600 Pa	0.4 < MI < 1
Tripathi et al. [85]	H = 400 μm; W = 200 μm	Ethanol, water D = 1.24 × 10^−9^ m^2^/s	ANSYS Fluent 14	M1 = 2,329,800 M2 = 2,447,975 M3 = 2,219,891	0.1 ≤ *Re* ≤ 100; 900 < *Pe* < 90,000	ΔP < 35,000 Pa	MI < 0.9
Tripathi et al. [84]	H = 400 μm; W = 200 μm; 12.24 mm ≤ L ≤ 31.65 mm	Ethanol, water D = 1.24 × 10^−9^ m^2^/s	ANSYS Fluent 18.1	M < 3,500,000	1 ≤ *Re* ≤ 100; 900 < *Pe* < 90,000	ΔP < 11,000 Pa	0.1 < MI < 0.9
Yuan et al. [87]	Width = 60 μm; H = 150 μm; L < 5000 μm	Ethanol, water	COMSOL Multiphysics 5.4; Ansys Fluent 15.0	1,250,000 ≤ M ≤ 1,850,000	0.1 ≤ *Re* ≤ 50; 52 < *Pe* < 7600	0.08 kPa < ΔP < 80 kPa	0.8 < MI < 1
Bahrami and Bayareh [26]	W = H = 600 μm; L < 80 mm	Water–water	ANSYS Fluent	27,300 < M < 9,870,000	0 ≤ *Re* ≤ 100	0.1 kPa < ΔP < 45 kPa	0.1 < MI < 1
Niu et al. [32]	W = H = 600 μm; L = 10 mm	Water–water with concentration from 0 to 1 mol/m^3^	COMSOL Multiphysics 5.4	M1 = 33,976 M2 = 44,455 M3 = 62,207 M4 = 75,378	0.01 < *Re* < 50; 9 < *Pe* < 46,000	5 Pa < ΔP < 90,000 Pa	0.2 < MI ≤ 1
Fatima and Shakaib [92]	W = H = 400 μm; L = 6000 μm	Water–water with concentration from 0 to 1 mol/m^3^	ANSYS Fluent	19-	20 ≤ *Re* ≤ 260	*ΔP* < 6000 Pa	MI < 0.1; 0.8 < SI < 0.90
Karthikeyan et al. [89]	W = 200 μm; L = 16.5 mm	Water–water with concentration from 0 to 1 mol/m^3^	COMSOL Multiphysics 6.0	M1 = 40,948 M2 = 55,750 M3 = 65,516 M4 = 137,008	-	ΔP < 1400 Pa	0.4 < MI < 1

**Table 4:** Analytical and empirical expressions for MI available in literature.

Author	Expression
Engler et al. [99]	$MI=1-\sqrt{\frac{\sigma_{M}^{2}}{\sigma_{M,max}^{2}}}$; $\sigma_{M}^{2}=\frac{1}{N}\sum_{i=1}^{N}{\left(C_{i}-\bar{C_{M}}\right)}^{2}$;
Rudyak et al. [34]	$MI=\frac{113.2}{1+0.037{Pe}^{0.7}}$
Tseng et al. [52]	$MI=1-\frac{1}{\bar{C_{M}}}\sqrt{\frac{1}{N}\sum_{i=1}^{N}{\left(C_{i}-\bar{C_{M}}\right)}^{2}}$
Roudgar et al. [55]	$MI=1-\sqrt{\frac{1}{N}\sum_{i=1}^{N}{\left(\frac{v_{i}C_{i}}{v\bar{C_{M}}}-1\right)}^{2}}$

**Table 5:** Analytical and empirical expressions for pressure drop available in the literature.

Author	Expression
Kirby, B.J. [101]	$∆P=\frac{8\mu QL}{\pi R^{4}}$
Howell and Weathers [102]	$∆P=f_{D}\cdot \frac{\rho }{2}\cdot \frac{{v_{avg}}^{2}}{D_{H}}\cdot L$; $D_{H}=4\frac{A}{P}$; $f_{D}=\frac{64}{Re}$
Babu et al. [90]	$∆P=\frac{1}{2}\xi \rho v^{2}$; $f_{D}=\frac{D_{H}}{2L}\xi$
Khaydarov et al. [70]	$∆P=0.0095{Re}^{2}+0.5508Re$
Hosseini and Rahimi [76]	λ=2∆PLDρv2=f+kDL

**Table 6:** Comparison of pressure drop variation in different categories of micromixers available in the literature if the micromixer were a straight microchannel and corresponding mixing index predicted by numerical simulations or measured by experimental measurements.

Reference	Passive Micromixer	Category	*Re*	% Increase Pressure Drop Compared with Straight Channel	Mixing Index
Okuducu and Aral [41]	TJM	1	40	51%	25%
Lotfiani and Rezazadeh [81]	YJM	1	100	164%	58%
Mahmud et al. [83]	YUM	2	50	83%	51%
Khaydarov et al. [70]	LTFMSM	2	52	69%	25%
Mahmud et al. [83]	HCM	2	50	163%	94%
Okuducu and Aral [41]	CSCRM	3	40	337%	80%
Niu et al. [32]	SBM	3	50	561%	53%
Niu et al. [32]	CSBM	3	50	2797%	98%
Niu et al. [32]	ESBM	3	50	4767%	94%
Lotfiani and Rezazadeh [81]	TLM	4	100	883%	81%
Borgohain et al. [71]	TJMRWP	3	51	430%	85%
Yuan et al. [87]	ASZMMM	3	50	225%	100%
Tripathi et al. [84]	YJM	2	50	150%	90%
Wang et al. [57]	TSM	4	50	110%	75%
Ruijin et al. [40]	BM	4	20	101%	60%

**Table 7:** Number of mesh elements used for the numerical studies by different authors.

Author	Total Mesh	Smallest Element, μm
Chen et al. [74]	155,794	5
Amar et al. [77]	643,203	6
Mahmud et al. [83]	7.54 × 10^5^	20
Okuducu and Aral [78]	1.45 × 10^6^	5
Tripathi et al. (a) [84]	1,635,434	11
Niu et al. [32]	62,207	20
Yuan et al. [87]	3.87 × 10^6^	5

## Abbreviations

*Alphabetical letters*		
A	Total Available Area for the Fluid Flow	m^2^
∆b	Buoyancy term	m·s^−2^
Ca	Capillary Number	-
C_i_	Concentration at the ith point	mol·m^−3^
$\bar{C_{M}}$	Average Concentration of the N points	mol·m^−3^
D	Diameter	m
*D_AB_*	Diffusivity of species A in species B	m^2^·s^−1^
De	Dean number	-
D_H_	Hydraulic Diameter of the Geometry in which the Flowing Fluid	m
*d_h_*	Hydraulic diameter of the flowing fluid	m
*f*	fanning Friction Factor	-
*f_D_*	Darcy’s Friction Factor	-
L	Charasteristic length	m
MI	Mixing Index	-
N	Total Number of points used in the Calculation of the Mixing Index	-
Nu	Nusselt number	-
P	Total Available Perimeter for the Fluid Flow	M
*Pe*	Peclet Number	-
∆P	Pressure Drop	Pa
Pr	Prandtl number	-
Q	The Volumetric Flow rate of the Fluid	m^3^·s^−1^
R	radius	m
*R_c_*	Radius of curvature	m
*Ri*	Richardson number	-
*Re*	Reynolds Number of the Flowing Fluid	-
*Sc*	Schmidt number	-
*Sh*	Sherwood number	-
*St*	Stanton number	-
∆*U*	Difference in linear velocity	m·s^−1^
*U*	Linear velocity	m·s^−1^
*K*	Local Loss Factor	-
*v_qvg_*	Average Velocity of the Flowing Fluid	m·s^−1^
*v_i_*	Velocity at the ith point	m·s^−1^
*Greek Alphabets*		
*λ_1_*	Overall Friction Factor	-
*λ*	Mean free path in definition of Knudsen number	-
*μ*	Dynamics Viscosity of the Flowing Fluid	Pa·s
*ξ*	Effective Friction Factor	-
*ρ*	Density of the Flowing Fluid	kg·m^−3^
*σ_M_*	Standard Deviation of the Concentration Field of the Mixture	-
*σ_M,max_*	Maximum Standard Deviation of the Concentration Field of the Mixture	-
*σ*	Surface tension, Nm^−1^	
